# Effect of Long-term Nicotine Replacement Therapy vs Standard Smoking Cessation for Smokers With Chronic Lung Disease: A Randomized Clinical Trial

**DOI:** 10.1001/jamanetworkopen.2018.1843

**Published:** 2018-09-07

**Authors:** Edward F. Ellerbeck, Nicole Nollen, Tresza D. Hutcheson, Milind Phadnis, Sharon A. Fitzgerald, James Vacek, Matthew R. Sharpe, Gary A. Salzman, Kimber P. Richter

**Affiliations:** 1 Department of Preventive Medicine and Public Health, University of Kansas Medical Center, Kansas City, Missouri; 2 Department of Biostatistics, University of Kansas Medical Center, Kansas City, Missouri; 3 Department of Cardiology, University of Kansas Medical Center, Kansas City, Missouri; 4 Department of Pulmonary and Critical Care Medicine, University of Kansas Medical Center, Kansas City, Missouri; 5 Department of Internal Medicine, University of Missouri-Kansas City, Kansas City, Missouri

## Abstract

**Importance:**

Smokers with chronic obstructive pulmonary disease (COPD) have particular difficulty quitting. Long-term nicotine replacement therapy (LT-NRT) might offer a strategy for reducing harm from cigarettes and provide a pathway for later cessation.

**Objective:**

To compare the effect of LT-NRT vs standard smoking cessation (SSC) on exposure to cigarette smoke, harm related to smoking, and cessation among smokers with COPD.

**Design, Setting, and Participants:**

This unblinded, randomized clinical trial recruited smokers who self-reported a diagnosis of COPD at any level of readiness to quit from May 23, 2014, through November 30, 2015. The 12-month follow-up was completed December 6, 2016. Patients were recruited at a clinical research unit of an academic medical center. Analysis was based on intention to treat and performed from March 8 through November 30, 2017.

**Interventions:**

Standard smoking cessation treatment included 10 weeks of NRT and 4 follow-up counseling sessions for those willing to make a quit attempt. Long-term NRT included 12 months of NRT and 6 follow-up counseling sessions regardless of initial willingness to quit. Overall, 198 patients were randomized to SSC, and 197 were included in the primary analysis; 200 patients were randomized to LT-NRT, and 197 were included in the primary analysis.

**Main Outcomes and Measures:**

The primary outcome was 7-day abstinence verified by carbon monoxide (CO) levels at 12 months. Secondary outcomes included cigarettes smoked per day (CPD), exposure to CO, urinary excretion of 4-methylnitrosamino-1-3-pyridyl-1-butanol (NNAL) (a smoking-related carcinogen), and adverse events.

**Results:**

Among 398 patients who were randomized (59.8% female; mean [SD] age, 56.0 [9.3] years), the mean (SD) CPD was 23.1 (12.3). Twelve-month follow-up was completed by 373 participants (93.7%), and 394 (99.0%) were included in the primary analysis. At 12 months, CO-verified abstinence occurred in 23 of 197 participants (11.7%) in the SSC arm and 24 of 197 (12.2%) in the LT-NRT arm (risk difference, 0.5%; 95% CI, −5.9% to 6.9%). Continuing smokers in the SSC and LT-NRT arms had similar, significantly reduced harms caused by smoking, including cigarette consumption by 12.4 and 14.5 CPD, respectively, exhaled CO level by 5.5 and 7.8 ppm, respectively, and mean urinary NNAL excretion by 21.7% and 23.0%, respectively. In multivariate analyses, continuing smokers with greater adherence to NRT experienced less reduction in NNAL exposure. The frequency of major adverse cardiac events was similar in both groups.

**Conclusions and Relevance:**

Similar rates of cessation and similar reductions in exposure to tobacco smoke resulted with LT-NRT and SSC. Among continuing smokers, ongoing use of NRT was not associated with reductions in smoke exposure.

**Trial Registration:**

ClinicalTrials.gov Identifier: NCT02148445

## Key Points

**Question:**

Compared with standard cessation approaches, can long-term nicotine replacement therapy lead to higher rates of cessation or reductions in carcinogen exposure among smokers with chronic obstructive pulmonary disease?

**Findings:**

In this randomized clinical trial of 398 smokers with chronic obstructive pulmonary disease, 23 of 197 (11.7%) receiving a standard smoking cessation intervention had quit at 12 months, compared with 24 of 197 (12.2%) receiving long-term nicotine replacement therapy. Both groups had comparable reductions in carcinogen and smoke exposure.

**Meaning:**

Long-term nicotine replacement therapy provides an option for smokers with chronic obstructive pulmonary disease but does not result in greater rates of cessation or harm reduction.

## Teaser

This randomized clinical trial compares the effect of long-term nicotine replacement therapy vs standard smoking cessation on exposure to cigarette smoke, harm related to smoking, and smoking cessation among smokers with chronic obstructive pulmonary disease.

## Introduction

Most chronic obstructive pulmonary disease (COPD) in the United States can be attributed to smoking, and smoking cessation should be the first step for treating the disease.^1^ Compared with those who quit, patients with COPD who continue to smoke have higher mortality^2^ and more rapid decline in pulmonary function.^3^ These persistent smokers have particularly high levels of nicotine dependence^4,5,6^ and lower self-efficacy for quitting^7^ and find it more difficult to quit.^8^ Despite the effectiveness of counseling and smoking cessation pharmacotherapy,^9^ smokers with COPD are less likely than other smokers to succeed in their quit attempts.^10^

Better strategies are needed for treating patients with COPD who continue to smoke. Long-term nicotine replacement therapy (LT-NRT) as a pathway to cessation or as a method for harm reduction might be an option. Most smokers trying to quit fail in the attempt and quickly resume smoking.^11^ These smokers might benefit from continued pharmacotherapy to reduce harm from smoking or support subsequent quit attempts. Several investigators^12,13,14^ have looked at extended treatment with NRT for 6 to 12 months after a quit attempt. These studies have had mixed results, with 2 showing improvements in smoking cessation at 6 months that were not sustained at 1 year.^13,14^ Another study^12^ failed to demonstrate differences at 6 months but showed improvements in cessation 12 to 18 months after study enrollment. None of these studies examined smoke exposure or harm reduction among continuing smokers, and none of these studies were focused on patients with COPD.

Heavy smokers, such as those with COPD, may be particularly interested in gradual tapering or cutting down the number of cigarettes smoked (reduce-to-quit strategy) rather than quitting right away.^15^ A meta-analysis of 7 trials using NRT among smokers not willing to quit right away^16^ found a reduce-to-quit approach doubled the chances of eventually being able to quit. The actual effect size in these studies, however, was small (number needed to treat = 29).

Prior studies using extended NRT to support a cessation attempt or to help smokers reduce to quit were not focused on patients with COPD and did not examine the effect of ongoing NRT on smoke exposure among smokers unable to quit. The purpose of this study was to compare a standard smoking cessation (SSC) program with LT-NRT provided to smokers with COPD regardless of their readiness to quit and examine the effect of long-term NRT on smoking cessation and harm reduction.

## Methods

This randomized, unblinded clinical superiority trial compared the efficacy of LT-NRT vs SSC for smokers with COPD and followed the Consolidated Standards of Reporting Trials (CONSORT) reporting guidelines. Recruitment occurred from May 23, 2014, through November 30, 2015, with final follow-up completed by December 6, 2016. Consistent with the principles of the Patient-Centered Outcomes Research Initiative,^17^ patients and stakeholders (ie, members of the community with a stake or interest in specific health care issues, including clinicans, advocacy groups, and policy makers) were engaged during all phases of research. The study protocol appears in Supplement 1. The study was reviewed and approved by the institutional review board at the University of Kansas Medical Center, Kansas City, Missouri. Trained research assistants explained the study procedures to prospective participants and obtained written consent.

### Participants and Setting

Potential participants were identified by querying electronic health records^18^ at 2 academic medical centers; they were mailed a recruitment letter followed by telephone calls. Participants were also referred by other participants and health care clinicians in the Kansas City, Missouri, metropolitan region, including physicians, respiratory therapists, and nurses.

Participants were considered eligible, regardless of their willingness to quit, if they were 18 years or older, smoked 5 or more cigarettes per day on at least 25 of the last 30 days, spoke English or Spanish, reported physician-diagnosed COPD, and were willing to complete study requirements. Smokers were excluded if they had a terminal medical condition, would be pregnant or breastfeeding in the next year, resided in a long-term care facility that restricted smoking, exhibited severe cognitive impairment, had another household member enrolled in the study, had no home address, or had been hospitalized with a heart attack, experienced an irregular heartbeat, or reported increasing angina in the past 30 days.

### Randomization

After providing informed consent and completing baseline measures, participants were randomly assigned in a 1:1 ratio to the SSC or the LT-NRT study arm. Randomization occurred at the participant level in permuted blocks of 6 using the on-board randomization module in the research electronic data capture (REDCap) database system, which conceals allocation before enrollment.^19^

### Interventions

#### Standard Smoking Cessation

Counseling for SSC was designed to emulate a typical smoking cessation program, such as a state-sponsored tobacco quitline.^11^ During the baseline session, counselors used motivational strategies to increase readiness to quit. Participants who set a quit date received a personalized quit plan, incorporating the use of NRT, as outlined below. Those not ready to quit were included in the study but did not receive active treatment unless they set a quit date within 6 weeks of randomization. Participants in the SSC arm who set a quit date were proactively contacted for telephone counseling 1, 3, 6, and 10 weeks after the baseline visit. The mean (SD) length of the counseling sessions was 15.4 (7.0) minutes. In addition, they received a 10-week supply of combination NRT to start on their quit date.

#### Long-term NRT

Participants randomized to the LT-NRT arm completed baseline counseling that focused on use of NRT and interest in quitting. Counselors helped participants develop personalized quit plans or, for those who declined to set a quit date, cigarette reduction goals. Regardless of whether participants set a quit date, follow-up telephone counseling was provided at 1, 3, and 6 weeks and 9 months after baseline and in person at months 3 and 6. The mean (SD) length of the counseling sessions was 15.5 (7.3) minutes. Follow-up counseling provided support for NRT adherence and addressed relapse prevention for quitters or cigarette reduction and cessation planning for continuing smokers. Participants in the LT-NRT arm started their NRT immediately and received a new supply of NRT every 3 months.

### Combination NRT (Both Arms)

Combination NRT included nicotine patches plus 2 mg of nicotine gum and/or lozenges. The dose of nicotine patches (14-42 mg) provided to participants was based on their current cigarette consumption^11,20^ and consistent with combination NRT regimens previously shown to be effective and safe.^21,22^ Participants were instructed to use NRT for their entire treatment course even if they continued smoking. At each counseling session, the counselor assessed NRT use and adverse effects. Counselors worked with participants to mitigate adverse effects, improve medication adherence, and adjust dosages to minimize symptoms of withdrawal or craving.

### Outcomes, Measures, and Follow-up

Participants completed assessments at baseline and 3, 6, and 12 months after enrollment at a clinical research unit and received $50 for each visit. Data were entered into a REDCap database with built-in validity checks.^19^ At baseline, we assessed demographics, smoking characteristics, and health status^23,24,25,26,27,28^ (Table 1). Race and ethnicity were self-reported by participants based on predefined federal classifications. Use of NRT was assessed via 3-day recall at 3, 6, and 12 months.^29^

**Table 1.** Baseline Characteristics of Study Participants

Characteristic	Study Arm		
	All Participants (N *=* 398)	LT-NRT (n* =* 200)	SSC (n* =* 198)
Age, mean (SD), y	56.0 (9.3)	55.6 (9.9)	56.3 (8.7)
Female, No. (%)	238 (59.8)	127 (63.5)	111 (56.1)
Race, No. (%)			
White	277 (69.6)	145 (72.5)	132 (66.7)
African American	113 (28.4)	52 (26.0)	61 (30.8)
Other^a^	8 (2.0)	3 (1.5)	5 (2.5)
Hispanic ethnicity, No. (%)	13 (3.3)	5 (2.5)	8 (4.0)
Employed, No. (%)	80 (20.1)	40 (20.0)	40 (20.2)
Educational attainment of high school graduation or less, No. (%)	202 (50.8)	101 (50.5)	101 (51.0)
Any health insurance, No. (%)	333 (83.7)	167 (83.5)	166 (83.8)
Medicaid, No. (%)	169 (42.5)	80 (40.0)	89 (44.9)
Living status, No. (%)			
Lives alone	133 (33.4)	72 (36.0)	61 (30.8)
Other smokers living in household	155 (38.9)	77 (38.5)	78 (39.4)
Only nonsmokers in the home	110 (27.6)	51 (25.5)	59 (29.8)
CPD, mean (SD)^b^	23.1 (12.3)	24.0 (12.6)	22.1 (11.9)
Exhaled CO level, mean (SD), ppm	22.5 (13.8)	22.7 (14.5)	22.2 (13.1)
Smoke first cigarette within 30 min of waking, No. (%)	374 (94.0)	188 (94.0)	186 (93.9)
Heavy Smoking Index score ≥4, No. (%)^c^	255 (64.1)	129 (64.5)	126 (63.6)
Confidence to Quit score, mean (SD)^d^	6.6 (2.8)	6.6 (2.9)	6.6 (2.7)
Planning to quit in next 30 d, No. (%)^b^	326 (81.9)	169 (84.5)	157 (79.3)
No. of quit attempts in the past 12 mo, mean (SD)^b^	2.1 (4.7)	1.9 (4.3)	2.2 (5.1)
Previous use of e-cigarette, No. (%)	280 (70.4)	142 (71.0)	138 (69.7)
Use of e-cigarette in past 7 d, No. (%)	51 (12.8)	27 (13.5)	24 (12.1)
Prior use of NRT to cut down, No. (%)	149 (37.4)	76 (38.0)	73 (36.9)
Any prior use of cessation pharmacotherapy, No. (%)	324 (81.4)	163 (81.5)	161 (81.3)
Nicotine patch	234 (58.8)	116 (58.0)	118 (59.6)
Nicotine gum	110 (27.6)	56 (28.0)	54 (27.3)
Nicotine lozenge	43 (10.8)	21 (10.5)	22 (11.1)
Bupropion	86 (21.6)	44 (22.0)	42 (21.2)
Varenicline tartrate	162 (40.7)	88 (44.0)	74 (37.4)
Bupropion and nicotine patch in combination	11 (2.8)	4 (2.0)	7 (3.5)
Nicotine patch and short-acting therapy in combination	32 (8.0)	16 (8.0)	16 (8.1)
Spirometry, FEV_1_:FVC ≤0.70 (actual), No. (%)	242 (60.8)	119 (59.5)	123 (62.1)
CAT score >20, No. (%)^e^	231 (58.0)	124 (62.0)	107 (54.0)
≥2 COPD exacerbations in the past year, No. (%)^f^	76 (19.1)	30 (15.0)	46 (23.2)
Rate current health as fair or poor, No. (%)	231 (58.0)	118 (59.0)	113 (57.1)
Diabetes, No. (%)	132 (33.2)	69 (34.5)	63 (31.8)
Heart disease, No. (%)	87 (21.9)	48 (24.0)	39 (19.7)
BMI, mean (SD)	30.0 (8.4)	30.5 (8.7)	29.6 (8.0)
Anxiety, GAD-2 score ≥3,^g^	166 (41.7)	88 (44.0)	78 (39.4)
Depression, PHQ-2 score ≥3, No. (%)^h^	133 (33.4)	70 (35.0)	63 (31.8)

Abbreviations: BMI, body mass index (calculated as weight in kilograms divided by height in meters squared); CAT, COPD Assessment Test; CO, carbon monoxide; COPD, chronic obstructive pulmonary disease; CPD, cigarettes smoked per day; e-cigarette, electronic cigarette; FEV_1_, forced expiratory volume in the first second of expiration; FVC, forced vital capacity; GAD-2, 2-item General Anxiety Disorder Scale; LT-NRT, long-term nicotine replacement therapy; PHQ-2, 2-item Patient Health Questionnaire; SSC, standard smoking cessation.

^a^ Includes Native American, Alaskan Native, and Native Hawaiian or Pacific Islander.

^b^ Smoking history questions from Stage of Change Questionnaire.^23^

^c^ Scores range from 0 to 6, with 4 or greater indicating moderate to high nicotine dependence.^24^

^d^ Confidence to quit smoking scores range from 0 to 10.

^e^ Scores range from 0 to 40, with greater than 20 indicating high effect of COPD.^25,26^

^f^ Calculated by adding COPD-associated hospitalizations and emergency department visits in the year before baseline.

^g^ Scores range from 0 to 6, with 3 or greater indicating possible presence of general anxiety disorder.^27^

^h^ Scores range from 0 to 6, with 3 or greater indicating presence of depressive symptoms.^28^

The primary outcome was the 7-day point prevalence of smoking abstinence at 12 months confirmed by exhaled carbon monoxide (CO) levels of no greater than 10 ppm (except 1 participant at month 12 and 2 at month 6 who lived outside the area and were verified by proxy).^30^ Secondary cessation-associated outcomes included 6-month sustained abstinence (CO-verified point prevalence of abstinence at months 6 and 12) and cumulative number of 24-hour quit attempts.

Harm reduction outcomes included cigarettes smoked per day (CPD), exposure to CO, and carcinogen exposure as measured by 4-(methylnitrosamino)-1-(3-pyridyl)-1-butanol (NNAL) excretion. Concentrations of total cotinine, 3-hydroxycotinine, and total NNAL were measured in spot urine samples^31^ using fully validated liquid chromatography–mass spectrometry. Analytic procedures were based on established methods for measurement of cotinine, 3-hydroxycotinine,^32^ and NNAL.^33^ Limits of quantitation were 15 ng/mL for cotinine and 3-hydroxycotinine (to convert cotinine and 3-hydroxycotinine measures to nanomoles per liter, multiply by 5.675 and 5.203, respectively) and 30 pg/mL for NNAL. Values falling below the level of quantitation were imputed as half the lower limit of quantitation.^34^

Additional secondary outcomes related to physical function included change in pulmonary function (forced expiratory volume in the first second of expiration and forced vital capacity) from baseline to 12 months, respiratory symptoms as measured by the COPD Assessment Test (scores range from 0-40, with higher scores indicating greater effect of COPD),^25,26^ and cumulative number of respiratory-associated hospital admissions and emergency department visits. We assessed cardiovascular events during each follow-up assessment and confirmed events through medical record review. A Data Safety and Monitoring Committee provided oversight for safety monitoring procedures.

### Statistical Analysis

Data were analyzed from March 8 through November 30, 2017, using SAS software (version 9.4; SAS Institute, Inc). We generated descriptive statistics for each of the baseline measures. We used the χ^2^ test to assess for group differences in the primary and secondary cessation-associated outcomes. A 2-sided *t* test, assuming equal variances, was used to test for differences between groups in change in forced expiratory volume in the first second of expiration after testing for the equality of variances. We used generalized linear mixed models to test for differences between groups across time and for interactions between group and time for emergency department visits and hospitalizations, quit attempts, respiratory symptoms, expired CO level, and NNAL excretion.^35^ Measurements of NNAL excretion were adjusted for creatinine level^36^ and log-transformed. The correlation of multiple measurements from the same participant across time was modeled using heterogeneous, first-order, autoregressive structures. We used logistic and linear regression analyses to examine the association among participant characteristics, month 12 abstinence, and reduction in CO levels and NNAL excretion (eTables 1-3 in Supplement 2). Significance was defined as *P* < .05 with a 2-sided test.

For our primary outcome, we treated those with missing data as continued smokers. We conducted a variety of sensitivity analyses, including a completers-only analysis, an analysis assuming that all nonrespondents had quit smoking, and an analysis using a CO level cutoff of no greater than 5 ppm. We performed logistic regression analyses controlling for variables that were unbalanced across treatment arms at baseline. Our linear and generalized linear mixed models addressed missing data under the missing-at-random assumption using likelihood-based analyses.^37^

The sample size was based on the primary outcome. We estimated that 60% of participants in the SSC arm would be interested in quitting smoking at the time of recruitment^38^ and would receive active treatment and that the cessation rate in this group would be 10%. Previous trials have shown that LT-NRT, provided as part of a reduce-to-quit strategy, is associated with a 2-fold increase in smoking cessation among smokers not yet ready to quit.^16,39,40,41^ Among smokers ready to quit, LT-NRT has been associated with odd ratios for quitting of 1.70 to 1.81.^12,13,14^ Although prior studies of LT-NRT have primarily used a single form of NRT, combination therapy is associated with a 34% higher quit rate when compared with use of a single agent.^39^ Based on these data, we projected a 2-fold increase in cessation; a sample size of 398 participants provided a power of 80% to detect a 2-fold increase in cessation with a type I error of 5%.

## Results

Of 667 participants screened, 398 (238 women [59.8%] and 160 men [40.2%]; mean [SD] age, 56.0 [9.3] years) were randomized to the SSC (n = 198) or the LT-NRT (n = 200) arm (Figure). Participant characteristics across study arms were similar at baseline (Table 1). Most participants were white (277 [69.6%]) and not employed (318 [79.9%]). Participants had been diagnosed with COPD for a mean (SD) of 6.9 (7.5) years; 231 (58.0%) had a COPD Assessment Test score of greater than 20, indicating a high effect of symptoms from their COPD. Participants reported smoking a mean (SD) of 23.1 (12.3) CPD, with 374 (94.0%) smoking within 30 minutes of waking. Three hundred twenty-four participants (81.4%) had used 1 or more types of cessation pharmacotherapy in the past, 218 (54.8%) had made 1 or more quit attempts in the past year, and 326 (81.9%) indicated an interest in quitting within the next 30 days.

**Figure.**
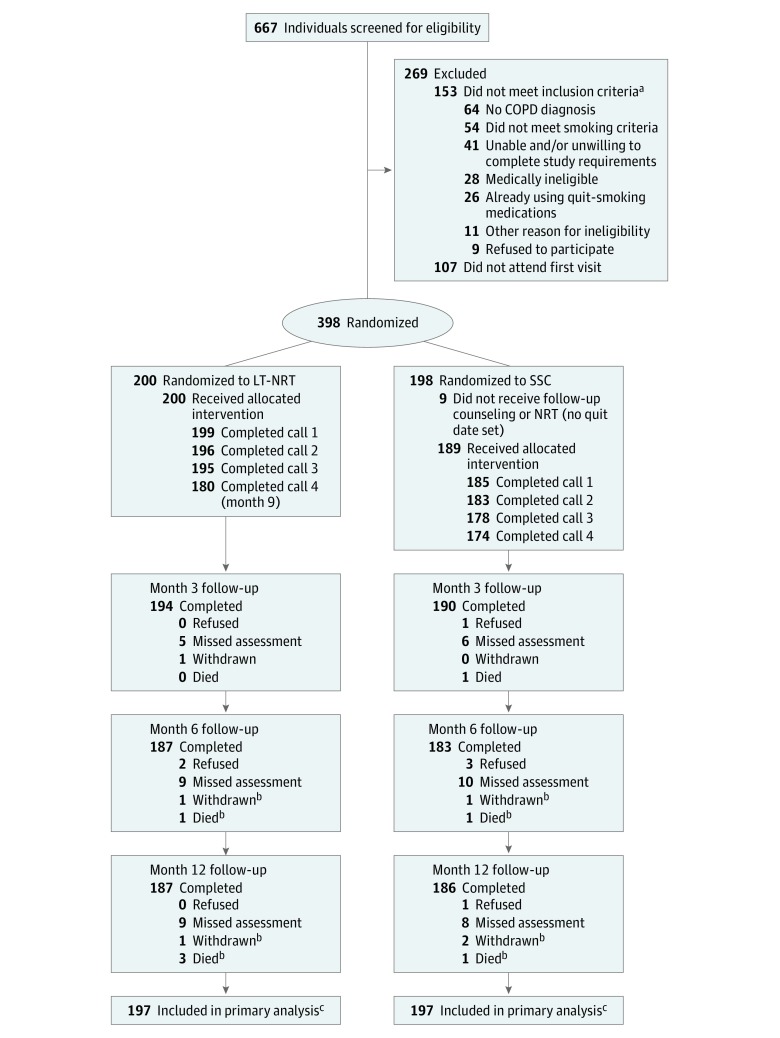
Study Flow (CONSORT) Diagram COPD indicates chronic obstructive pulmonary disease; LT-NRT, long-term nicotine replacement therapy; and SSC, standard smoking cessation. ^a^Includes multiple categories. ^b^Categories are cumulative. ^c^Participants who died were excluded from the analysis.

### Follow-up and Adherence to Therapy

Retention was comparable across treatment arms; 373 (93.7%) completed follow-up at 12 months and 394 (99.0%) were included in the primary analysis (Figure). Participants completed more than 95% of scheduled counseling sessions. At 3 months, 148 of 191 (77.5%) LT-NRT participants and 115 (60.8%) of 189 SSC participants reported using at least 1 type of NRT on a daily basis. At 6 months, these proportions were 144 of 185 (77.8%) and 48 of 181 (26.5%), respectively; at 12 months, 112 of 184 (60.9%) and 29 of 185 (15.7%), respectively. The LT-NRT participants reported a mean (SD) of 39.1 (11.2) weeks of nicotine patch use (n = 176) and 36.7 (13.3) weeks of nicotine gum or lozenge use (n = 176). The SSC participants reported a mean (SD) of 15.9 (9.9) weeks of nicotine patch use (n = 169) and 20.4 (12.4) weeks of nicotine gum or lozenge use (n = 170).

### Smoking Cessation

At 12 months, CO-verified 7-day abstinence (primary outcome) was confirmed in 24 participants (12.2%) in the LT-NRT arm and 23 (11.7%) in the SSC arm (risk difference, 0.5%; 95% CI, −5.9% to 6.9%) (intent-to-treat analysis with those missing data imputed as smokers) (Table 2). Sensitivity analyses provided similar results. Self-reported smoking cessation at 3, 6, and 12 months and 6-month sustained abstinence were not significantly different across treatment arms. In multivariate analyses, only increased age was associated with higher rates of cessation (odds ratio, 4.30; 95% CI, 2.25-8.19; *P* < .001) (eTable 1 in Supplement 2).

**Table 2.** Smoking Cessation Outcomes Between SSC and LT-NRT Arms

Outcome	Study Arm, No. (%) of Participants			Risk Ratio (95% CI)	Risk Difference (95% CI), %
	All (n = 394)	SSC (n = 197)	LT-NRT (n = 197)		
Primary outcomes at month 12					
Self-reported 7-d abstinence	53 (13.5)	25 (12.7)	28 (14.2)	1.12 (0.68 to 1.85)	1.5 (−5.2 to 8.3)
Biochemically verified 7-d abstinence^a^	47 (11.9)	23 (11.7)	24 (12.2)	1.04 (0.61 to 1.78)	0.5 (−5.9 to 6.9)
Secondary outcomes					
Sustained abstinence at 6 mo^a^^,^^b^	27 (6.9)	15 (7.6)	12 (6.1)	0.80 (0.38 to 1.67)	−1.5 (−6.5 to 3.5)
Self-reported 7-d abstinence at 3 mo	43 (10.9)	27 (13.7)	16 (8.1)	0.59 (0.33 to 1.06)	−5.6 (−11.7 to 0.6)
Biochemically verified abstinence at 3 mo^a^	39 (9.9)	25 (12.7)	14 (7.1)	0.56 (0.30 to 1.04)	−5.6 (−11.5 to 0.3)
Self-reported 7-d abstinence at 6 mo	48 (12.2)	28 (14.2)	20 (10.2)	0.71 (0.42 to 1.22)	−4.1 (−10.5 to 2.4)
Biochemically verified abstinence at mo 6^a^	44 (11.2)	25 (12.7)	19 (9.6)	0.76 (0.43 to 1.33)	−3.1 (−9.3 to 3.2)

Abbreviations: LT-NRT, long-term nicotine replacement therapy; SSC, standard smoking cessation.

^a^ Confirmed by exhaled carbon monoxide levels of no greater than 10 ppm, except 1 participant at month 12 and 2 at month 6 verified by proxy. Those who did not provide verification were treated as smokers.

^b^ Sustained abstinence defined as verified quit at 6-month and 12-month follow-up.

### Secondary Outcomes

Although participants’ respiratory function measurements remained unchanged (Table 3), both groups experienced similar improvements in respiratory symptoms over time, with the mean COPD Assessment Test score improving by 4.6 points in the LT-NRT arm and 3.6 points in the SSC arm. Similar numbers of participants in the 2 treatment arms had 1 or more respiratory-associated emergency department visits or hospitalizations during the 12 months of follow-up. Both groups reported similar frequency of quit attempts.

**Table 3.** Secondary Outcomes by Arm and Time Point^a^

Outcome by Study Arm	Baseline		Month 3		Month 6		Month 12		*P* Value		
	No.	Mean (SD)	No.	Mean (SD)	No.	Mean (SD)	No.	Mean (SD)	Group	Time	Group × Time
**All Participants**											
CPD											
SSC	197	22.1 (11.9)	189	7.9 (8.6)	182	8.1 (8.5)	186	8.5 (7.8)	.16	<.001	.05
LT-NRT	197	23.9 (12.5)	191	10.5 (8.6)	185	9.1 (9.5)	185	8.1 (8.3)			
CO level, ppm											
SSC	197	22.3 (13.1)	174	15.0 (12.6)	164	14.6 (11.9)	175	15.5 (11.1)	.75	<.001	.05
LT-NRT	197	22.8 (14.6)	185	17.4 (13.7)	167	15.4 (12.6)	176	13.8 (11.1)			
NNAL excretion, pg/mg of creatinine^b^											
SSC	197	322.4 (269.2)	188	279.0 (353.4)	180	178.2 (221.3)	183	190.4 (233.2)	.79	<.001	.39
LT-NRT	197	311.7 (301.6)	190	320.1 (350.6)	184	186.0 (236.2)	180	183.5 (223.9)			
No. of quit attempts of 24 h or longer^c^											
SSC	197	2.2 (5.1)	189	4.6 (6.5)	181	4.5 (6.9)	185	5.7 (8.6)	.55	NA	.40
LT-NRT	197	1.9 (4.3)	191	3.7 (5.5)	185	4.8 (9.0)	183	6.2 (11.3)			
Respiratory function, % of estimated FEV_1_^d^											
SSC	197	57.4 (18.6)	NA	NA	NA	NA	175	55.4 (19.3)	NA	NA	.42
LT-NRT	197	56.9 (21.1)	NA	NA	NA	NA	175	56.8 (19.8)			
Respiratory symptoms^e^											
SSC	197	21.1 (8.8)	189	17.8 (9.3)	181	17.9 (9.5)	185	17.5 (9.3)	.18	<.001	.27
LT-NRT	197	22.8 (8.3)	191	19.3 (8.7)	185	18.1 (8.5)	184	18.2 (9.4)			
Respiratory events^c^^,^^f^											
SSC	197	1.0 (1.7)	189	0.1 (0.4)	181	0.2 (0.8)	185	0.2 (0.7)	.69	NA	.06
LT-NRT	197	0.7 (1.4)	191	0.2 (0.5)	185	0.2 (0.7)	183	0.2 (0.6)			
**Continuing Smokers Only**^g^											
CPD											
SSC	174	22.1 (11.3)	166	8.7 (8.8)	159	9.1 (8.6)	163	9.7 (7.6)	.21	<.001	.05
LT-NRT	173	23.8 (12.2)	167	11.4 (8.5)	161	9.9 (9.5)	161	9.3 (8.3)			
CO level, ppm											
SSC	174	22.9 (13.3)	152	16.4 (12.6)	142	16.0 (11.7)	153	17.4 (10.6)	.74	<.001	.08
LT-NRT	173	23.6 (15.1)	161	18.8 (13.2)	143	17.0 (12.5)	152	15.8 (10.7)			
NNAL excretion, pg/mg of creatinine^b^											
SSC	174	328.1 (267.1)	165	314.6 (373.2)	158	219.8 (236.3)	160	257.0 (234.8)	.96	<.001	.77
LT-NRT	173	321.4 (305.6)	166	337.6 (363.8)	160	217.1 (251.8)	156	247.5 (230.6)			

Abbreviations: CO, carbon monoxide; CPD, cigarettes smoked per day; FEV_1_, forced expiratory volume in first second of expiration; LT-NRT, long-term nicotine replacement therapy; NA, not applicable; NNAL, 4-(methylnitrosamino)-1-(3-pyridyl)-1-butanol; SSC, standard smoking cessation.

^a^ Displays raw means for actual respondents at each time point; *P* values are based on model-based means for repeated-measure analyses.

^b^ Reported as geometric means and SD around the geometric mean calculated using delta method.

^c^ Measured as the number reported in the past year at baseline, the past 3 months at months 3 and 6, and the past 6 months at month 12.

^d^ The *P* value based on 2-sided *t* test difference from baseline to month 12 and includes 175 participants per arm who had data at both points.

^e^ Measured using the COPD Assessment Test. Scores range from 0 to 40, with higher scores indicating greater symptom severity.

^f^ Included emergency department visits and hospitalizations.

^g^ Analyses exclude all participants who were verified as quit at month 12.

At 12 months, among the participants who continued to smoke, both groups reported similar reductions relative to baseline in self-reported CPD (LT-NRT group, −14.5; SSC group, −12.4 CPD), expired CO level (LT-NRT group, −7.8 ppm; SSC group, −5.5 ppm), and NNAL excretion (LT-NRT group, −23.0%; SSC group, −21.7%). During the 12-month follow-up, these changes were significantly different from baseline, but did not differ significantly between groups (Table 3). In multivariate analyses, baseline cotinine levels (exponentiated β coefficient, 0.80; 95% CI, 0.67-0.97; *P* = .04), depressive symptoms (exponentiated β coefficient, 0.82; 95% CI, 0.68-0.98; *P* = .02), and daily use of NRT at month 12 (exponentiated β coefficient, 0.96; 95% CI, 0.92-1.00; *P* = .03) were inversely correlated with changes in NNAL levels (eTable 2 in Supplement 2). We found no significant association between participant characteristics or NRT use and changes in CO level (eTable 3 in Supplement 2).

### Adverse Events

During the course of the study, 17 major adverse cardiac events occurred, including 1 death. Nine events occurred in the SSC group and 8 in the LT-NRT group. Six events occurred while participants were receiving NRT and 11 occurred while not receiving NRT. Three noncardiac deaths were attributed to COPD complications, lung cancer, and pulmonary aspiration.

## Discussion

In this study of patients who continued to smoke despite being diagnosed with COPD, LT-NRT led to comparable rates of smoking cessation at 12 months compared with a traditional, NRT-supported, smoking cessation program. Long-term NRT and SSC were associated with modest rates of smoking cessation at 12 months (approximately 12%), highlighting the difficulty in achieving cessation in this group of smokers at high risk for treatment failure.

Although we tried to enroll smokers at all stages of readiness to quit, given the large number of patients in this study interested in quitting, our study has less in common with reduce-to-quit studies^16^ and more in common with prior studies of extended treatment with NRT for patients willing to quit.^12,13,14^ In 2 of these studies of extended treatment, Schnoll et al^13,14^ compared 8 weeks of NRT with 24 to 52 weeks of extended NRT treatment. In both trials, extended NRT therapy was associated with significantly higher abstinence rates at 24 weeks, but abstinence declined over time and, by week 52, abstinence was similar across treatment arms.^13,14^ Although our study failed to confirm the effect of extended NRT on abstinence at week 24, we observed a similar lack of benefit from extended treatment at week 52.

Another study, however, demonstrated long-term differences in outcomes associated with extended treatment.^12^ Joseph and colleagues^12^ compared 8 vs 48 weeks of NRT treatment. They found that point prevalence of abstinence at 6 months was virtually identical in both groups, but at 18 months, those receiving the extended treatment had significantly higher 6-month prolonged abstinence (adjusted odds ratio, 1.74). However, Joseph et al^12^ attempted to contact smokers every 2 to 4 weeks throughout the course of the study and engage them in new quit attempts if they were still smoking. Although abstinence rates declined from months 6 to 12 in the 2 studies of extended therapy by Schnoll et al,^13,14^ abstinence rates in the study by Joseph et al^12^ increased during the same period. In our study, where recipients of long-term NRT received counseling at months 6 and 9, confirmed abstinence increased from 9.6% at month 6 to 12.2% at month 12, whereas a slight decline in abstinence occurred during the same period for recipients of SSC. Together these findings suggest that the benefits of extended pharmacotherapy may depend on ongoing behavioral support and re-engagement of continuing or relapsed smokers in new cessation attempts. This conclusion is further supported by a previous study of long-term disease management for smoking cessation in which repeated engagement of smokers at 6-month intervals also demonstrated progressively increasing rates of cessation over time.^38^

Long-term NRT and SSC resulted in comparable levels of harm reduction among patients who continued to smoke. Persistent smokers in both study arms reported similar reductions of 62% to 66% in CPD, 30% to 39% in expired CO levels, and 19% to 30% in NNAL excretion; these reductions represented statistically significant changes from baseline. As seen in other studies, the reductions in CPD in our study exceeded the reductions in biological markers of cigarette exposure.^42,43^ Nevertheless, these reductions may be clinically significant. Reductions of 50% or more in CPD have been linked to improvements in cardiovascular risk factors, respiratory symptoms,^43^ and lung cancer risk.^44^ More importantly, reductions in smoking appear to support future quit attempts and ultimate cessation.^15^

Although participants who continued to smoke in both arms of this study reduced their cigarette consumption, LT-NRT was not the major factor in achieving these reductions. Indeed, continuing smokers who reported ongoing use of NRT at month 12 actually had less reduction in their NNAL excretion than those that had stopped using their NRT. This finding is consistent with other evidence that supplemental nicotine alone is not associated with reduced carcinogen exposure among continuing smokers^45^ and that addiction-associated factors (eg, sensory and environmental stimuli) other than nicotine play a major role in cigarette dependence in patients with COPD.^46^

This study adds to existing research establishing the safety of combination and higher-dose NRT among patients with multiple comorbidities,^47,48^ even if they continue to smoke. Although many participants continued to smoke while receiving high dosages of supplemental NRT, major adverse cardiovascular events were rare and appeared to have no association with the use of NRT.

The recruitment strategies used in this study highlight the potential value of proactive outreach and engagement of smokers with COPD. Our proactive queries of electronic health records provided lists of patients with COPD who were still smoking. We reached out to these smokers through direct mailings and follow-up telephone calls and were able to engage a large proportion of these patients in smoking cessation attempts.

The low overall rate of cessation in this study suggests the need for better strategies to help patients with COPD quit smoking. Future research could examine repeated interventions over time^38^ or modifications in therapy after an initial treatment failure.^49^ Extended treatment with other agents or delivery devices could also be tested. We chose to study combination NRT based on patient concerns about varenicline tartrate and data suggesting that combination NRT was as effective as varenicline.^50^ Varenicline, however, may be more effective than NRT in reducing reinforcement from cigarettes and results in higher rates of delayed cessation.^51^ New oversight by the US Food and Drug Administration^52^ should facilitate research on the use of electronic cigarettes for smoking cessation, but our findings and those of others^45^ suggest that unless smokers quit completely, NRT, whether offered through patch, gum, lozenge, or electronic cigarette, may not result in meaningful harm reduction.

### Limitations

Less than two-thirds of the patients randomized to LT-NRT were still using NRT at month 12, whereas many SSC participants reported ongoing use of NRT at 6 and 12 months. In our multivariate model, ongoing use of NRT was not associated with CO-verified cessation (eTable 1 in Supplement 2); nevertheless, nonadherence in the LT-NRT group and ongoing use of NRT by SSC participants could have diminished our ability to identify a treatment effect. The intensity of treatment in the SSC group may have exceeded the intensity typically offered in clinical practice and may have further limited our ability to detect a difference. Our study was conducted among volunteers prepared to take part in a research study; it is not clear how offers of LT-NRT in actual clinical practice might affect the potential reach of smoking cessation; findings might be different among less motivated smokers. Finally, our study was powered to detect a 2-fold difference between treatment arms; we cannot exclude the possibility of a smaller effect size that might still be clinically significant.

## Conclusions

For smokers with COPD, LT-NRT did not provide any advantages over a traditional smoking cessation program in terms of smoking cessation. The traditional smoking cessation intervention had a shorter duration of treatment and would appear to be the preferred treatment for smokers ready to quit. Long-term NRT can lead to rates of cessation comparable to SSC programs and might provide an option for smokers not immediately willing to quit. However, in the absence of cessation, LT-NRT, even at high dosages, does not appear to affect smoke exposure any more than a failed cessation attempt.

## Notes

Supplement 1. **Trial Protocol**

Supplement 2. **eTable 1.** Bivariate and Multivariate Associations Between Participant Characteristics and CO-Verified 7-Day Point Prevalence of Abstinence at Month 12 **eTable 2.** Bivariate and Multivariate Associations Between Participant Characteristics and NNAL Reduction From Baseline to Month 12 **eTable 3.** Bivariate and Multivariate Associations Between Participant Characteristics and 50% Reduction in CO From Baseline to Month 12
