# Supplementary material for: Effect of Long-term Nicotine Replacement Therapy vs Standard Smoking Cessation for Smokers With Chronic Lung Disease: A Randomized Clinical Trial
Source: JAMA Netw Open. 2018 Sep 7;1(5):e181843. doi: 10.1001/jamanetworkopen.2018.1843 (PMC6324503; doi:10.1001/jamanetworkopen.2018.1843)
Supplement: Supplement 1. — Trial Protocol [file jamanetwopen-1-e181843-s001.pdf]

RESEARCH PROTOCOL

More than 400,000 Americans die each year from a smoking-related illness.[1] Of the more than 43 million smokers in the United States today, 1 in 2, or more than 20 million, will die prematurely due to their smoking habit. Approximately 1/3 of these patients will die from smoking-related complications of chronic obstructive disease (COPD).[2]

More than 15 million Americans currently suffer from COPD.[3] Mirroring the distribution of cigarette smoking, COPD disproportionately affects the poor and disadvantaged.[3] COPD can have a profound impact on quality of life, resulting in difficulty breathing, chronic cough, poor exercise tolerance, disability, hospitalizations, and death.[4,5] Smoking is the primary cause of COPD, and quitting smoking is at the cornerstone of COPD treatment. For patients with COPD, quitting smoking can reduce the risk of further lung deterioration as well as the risk of COPD-related hospitalizations, death, and disability.[6-8]

Although smoking cessation is strongly recommended for all patients with COPD, patients with COPD have a particularly high dependence on nicotine and find it extremely difficult to quit.[9] Despite the ongoing damage to their lungs, more than 39% of people suffering from COPD continue to smoke.[3] It's not that these smokers don't recognize the danger of their habit – they do.[10] The vast majority would like to quit,[11] and each year half try,[1] but only about 3% actually succeed in quitting.[12]

The current standard of care for smoking cessation in the United States asks smokers to quit completely on a selected quit date, ideally with the aid of a smoking cessation treatment such as varenicline, bupropion, or nicotine replacement therapy (NRT).[13] This approach is unrealistic and unsuccessful for the majority of smokers; they find the idea of quitting cold turkey daunting and don't even try.[14] At any given time only 10-20% of smokers are ready and willing to make a quit attempt. Even for the motivated smokers who are willing to quit right away, 80% fail in the attempt and quickly resume smoking.[13] Not surprisingly, many smokers would like to have alternatives to this 'cold turkey' approach; they are interested in gradual tapering or cutting down on the number of cigarettes that they smoke until they are ultimately able to quit.[15]

In this study, we seek to compare the current standard of care (smoking cessation) with long-term guided maintenance therapy on NRT. In this randomized controlled trial, smokers with COPD will be randomized to receive either: 1) a standard smoking cessation (SSC) intervention consisting of counseling and NRT for smokers ready to quit; or 2) long-term guided maintenance therapy (LT-NRT) in which all smokers, regardless of their immediate interest in quitting, are treated with NRT and encouraged to cut down on their smoking and quit more gradually. Evidence suggests that the LT-NRT approach will not only result in lower exposure to toxic cigarette smoke, but will also outperform SSC in terms of smoking cessation by a factor of 2:1.[16-19] If this holds true for patients with COPD, LT-NRT could dramatically expand treatment options for smokers with COPD and make treatment much more approachable to the millions of patients with COPD who continue to smoke but are intimidated by the idea of immediately quitting.

In addition to directly comparing the benefits of SSC versus LT-NRT, our study will perform a classification and regression tree (CART) analysis that will result in the development of a clinical decision tree.[20,21] This decision tree will provide smokers with estimates of their chances of quitting or significantly reducing their smoking based on their treatment choice and their own personal characteristics. This decision tree will facilitate the dissemination of the findings of this study as it can be posted on the web or incorporated into the patient's electronic health record.[22] This decision tree will help smokers and their clinicians choose a treatment that matches their individual characteristics and their own desired outcomes. Our study could also inform reimbursement policies related to smoking cessation and NRT by providing evidence of the utility of long-term NRT maintenance therapy.

Why focus on smokers with COPD? Although we could have conducted this study within any population of smokers, we chose to focus on smokers with COPD because they are particularly vulnerable to the adverse effects of smoking.[2] A study in patients with COPD is more likely to demonstrate differences in clinical outcomes that could capture the attention of policy makers.[4] Moreover, clinicians and stakeholders may be

more willing to adopt the LT-NRT intervention in COPD patients since the benefits of cutting back or quitting appear particularly salient in this group. (This supposition was confirmed by our stakeholders.) Nevertheless, our findings could have important implications for the entire population of smokers and could help advance treatment for the 43 million smokers in the United States.

In addressing cigarette smoking and COPD, we are actually addressing multiple chronic diseases at once. Cigarette smoking itself has been characterized as a chronic disease and cigarette smoking is directly related to heart disease, chronic lung disease, and multiple types of cancer.[13] Smoking increases the risk of developing diabetes and osteoporosis. It increases the risk of periodontal disease, peptic ulcer disease, impotence, and reproductive disorders.[23] Given the relationship of smoking to so many chronic conditions, smoking cessation has been identified as one of the most important and cost-effective preventive health interventions available.[24]

Explain how the results of the study would likely improve health care and outcomes (*Criterion 2*)

In this section, we will show how our proposed study is strongly grounded in research on the potential benefits of using combination NRT, using extended treatment for smoking cessation, and providing NRT treatment prior to actually quitting. We will show, however, that this research, for a variety of reasons has not yet been translated into clinical practice, reimbursement policies, or informed treatment choices for patients. We will show how our research is highly responsive to research priorities outlined by expert groups and well aligned with the needs and interests of patients. Perhaps most importantly, we will show how the findings of our research could be quickly disseminated and have a profound impact on the current treatment of smokers with COPD and improve outcomes for this high-risk population.

Benefits of combined NRT therapy

We selected combination NRT as our pharmacological treatment for this study because NRT is the smoking cessation treatment most commonly used by smokers and was strongly supported by our Patient Advisory Panel.[25] NRT has fewer side effects than either bupropion (Zyban) or varenicline (Chantix),[13] and combination NRT appears to be at least as effective as any of the other pharmacological treatment for smoking cessation.[13] Combination therapy with NRT involves combining a long-acting nicotine product (i.e. nicotine patch) with a short-acting nicotine product (e.g. nicotine gum or nicotine lozenge); combination therapy has been found to be more effective than using either agent alone (RR 1.34, 95% CI 1.18 to 1.51).[26] Combination therapy with NRT results in quit rates comparable to those seen with varenicline (Chantix) without some of the concerns related to side-effects.[13,27] When NRT is used as monotherapy (e.g. gum or patch alone), smokers typically receive a lot less nicotine than they get by smoking.[28] Not surprisingly, therefore, some highly dependent smokers appear to exhibit a 'dose-response relationship' with NRT with those receiving higher doses having higher quit rates.[26] By using combination therapy, participants in this study should receive dosing of therapeutic nicotine closer to what they procure through their smoking. The patch will provide a steady dose of nicotine that reduces withdrawal and craving while the gum or lozenge will allow the smoker to personally titrate the dose of nicotine to the dose that is most effective.[29] Although clinical practice guidelines recommend NRT combination therapy as a first-line treatment for smoking cessation,[13] most insurers do not cover it, and it has not been widely implemented in clinical practice.[30-32] Positive results from our study would provide strong support for changing the standard of care related to use of combination NRT in high risk patients.

Benefits of extended treatment

Current practice guidelines recommend providing NRT treatment for 6-14 weeks.[13] For smokers willing to make an immediate quit attempt, use of extended treatment has led to mixed results,[13,33,34] leading to recommendations by expert groups for more research on the benefits of extended therapy.[13] Medication adherence has been an issue with many studies on extended pharmacotherapy and could account for some of these mixed results. On the other hand, in studies of continuing smokers not willing to quit immediately, the use of extended NRT has led to a 2-fold increase in cessation compared to short term treatment.[17,19] Meanwhile, 8-10 weeks of NRT remains the treatment standard[35] and insurance programs such as Kansas Medicaid place tight limits on the duration on treatment. Extended treatment with NRT has not been studied in

patients with COPD. The findings from our study, therefore could have a profound impact on guideline recommendations and reimbursement policy related to duration of therapy with NRT in smokers with COPD.

Importance of adherence

Discussions on duration of use may be moot if adherence is not addressed. Pharmacotherapy adherence has been consistently linked to improved treatment outcomes, and adherence to the prescribed dose and treatment length is necessary to achieve maximum drug effect.[36,37] Low adherence rates may lead to underestimates of the potential effectiveness of NRT. When smokers who are trying to quit 'slip' and have a cigarette, they may feel that the NRT is not working and quit taking it. In reality, 'slips' while on NRT may be a sign that they should be raising the dose (e.g. using more nicotine gum).[28] Our counseling in both arms of this study will specifically address 'adherence support' thereby increasing the validity of our study findings.

Efficacy and safety of precessation treatment

Use of NRT while the patient is still smoking has a strong grounding in addiction theory. If nicotine receptors in the brain are already saturated with nicotine derived from patch or gum, then the nicotine obtained from cigarettes won't be able to provoke a strong 'reward' stimulus in the brain – thereby effectively 'extinguishing' the behavioral reinforcement from smoking.[38] This theory is supported by empiric data. Smokers using NRT spontaneously reduce the amount that they smoke – even if they aren't actively trying to quit.[39-41] Indeed, it appears that smokers receiving NRT adjust (reduce) the amount that they smoke in order to maintain their usual nicotine intake.[42] The result of this is that smokers who use NRT while still smoking not only reduce their exposure to cigarette smoke,[26] but are also twice as likely to quit smoking.[13,17-19]

Use of NRT while the patient is still smoking is safe.[43] While labels on NRT products in the US continue to caution smokers and clinicians about the potential hazards of using NRT while still smoking,[44] there is extensive evidence that the risk of harm is virtually non-existent.[45-47] Nicotine is a sympathomimetic drug that can increase heart rate, increase blood pressure and contribute to endothelial dysfunction, but the dose-response curve for these effects appears to be relatively flat, so that when someone is already smoking, adding additional nicotine appears to have little effect on the cardiovascular system.[48] In addition, clinical trials of NRT in patients that already have cardiovascular disease have revealed no increased risk with use of NRT compared to placebo.[46] Researchers and policymakers in the United Kingdom recently completed an extensive safety analysis on use of NRT while smoking.[17] They not only concluded that this practice was safe, but also officially approved the use of NRT for continuing smokers trying to reduce smoking prior to quitting. In the United States, however, NRT is still reserved for smokers willing to go 'cold-turkey' and quit immediately.

Differences in policies in Europe and the United States show that there is still clinical uncertainty related to the use of NRT to support smoking reduction. Guidelines developed in the United States reviewed the evidence on precessation use of NRT and, despite a more than two-fold increase in quit rates and no evidence of harm, felt that more evidence was still needed to make before making precessation NRT a guideline-based recommendation.[13] In particular, they were concerned that the selective inclusion of resistant smokers in prior studies made it difficult to translate findings to the broader population of smokers. Our study directly addresses this concern by enrolling all smokers with COPD in this study, regardless of their immediate interest in quitting. Furthermore our analytic plan will allow us to see if treatment efficacy differs for smokers with different levels of readiness to quit. Therefore, this study could have important ramifications for future iterations of smoking cessation guidelines. With mandates for coverage of preventive services in the Affordable Care Act, these changes in guidelines could, in turn, have important implications for insurance coverage of NRT treatment.

Matching patients with optimal treatment based on their own characteristics

Our classification and regression tree (CART) analysis will allow us to provide more personalized recommendations for smokers with COPD and facilitate dissemination. CART is an analytical approach used as an alternative to logistic regression for the identification of subgroups of individuals who may have differential response to treatment. It can be used to develop a decision tree to guide clinical decision making.[20,21] CART uses statistical methods to cluster individuals into different groups that have different outcomes related to the treatment.[21] Using CART, multiple individual characteristics (e.g., gender, cigarettes

per day (cpd), cotinine level, readiness to quit, treatment assignment, etc.) can be considered simultaneously and variables can be identified that efficiently separate individuals into groups that are more or less likely to experience a positive outcome (e.g., abstinence). These variables can then be incorporated into a decision tree that will show the predicted outcome (response to treatment) based on a patient's given characteristics. The total number of splits or branches in the resulting decision tree depends on the number of critical variables identified in the CART analysis. The complexity of the decision tree can adapt itself to the complexity of the data. As a nonparametric approach, CART does not require specification of the nature of the relationship between predictors and outcome or a priori assumptions about the true model.[21]

The decision trees developed using a CART analysis have good face validity and can be easily applied clinically – the user simply follows the branches of the decision tree that are relevant to the specific individual. CART models have been developed in many areas of healthcare,[20,21,49] including tobacco research.[50,51] We propose to employ the CART approach to calculate abstinence rates for different subgroups using clinically relevant variables identified by literature review and our patients and stakeholders. The resulting decision tree will allow any smoker with COPD estimates to use their own personal characteristics to estimate their chances of stopping smoking given a specific treatment choice (Aim 2). The probability of other relevant outcomes (e.g. risk of worsening lung function) can be calculated for each branch of the decision tree as well. The ease of use of this type of decision tree will allow ready dissemination via web or smartphone applications or incorporation into clinical decision algorithms within electronic medical records.[22]

Our proposed research is highly responsive to many of the research priorities identified in the *Clinical Practice Guideline for Treating Tobacco Use and Dependence*.[13] These guidelines identified the need for more research related to long-term medication use for smoking cessation and the need for more studies on the use of NRT prior to quitting smoking.[13] The guidelines further identified the need for more studies on treatment of highly dependent smokers, including studies on use of combination medication therapy. The guidelines also identified a need for more research on ways to give smokers greater hope and confidence in treatment opportunities and to make treatment of tobacco use more accessible to smokers (e.g. those not yet ready to quit). Our proposed research addresses each of these research priorities.

Demonstrate the technical merit of the application (*Criterion 3*)

Study design

The proposed study will use a randomized clinical trial design to *compare the effectiveness* of 1) traditional smoking cessation (SC) versus 2) long-term, guided maintenance treatment with NRT (LT-NRT). Participants in the SSC arm (n=199) who are interested in quitting smoking the next 30 days will be offered 5 sessions of smoking cessation counseling over 12 weeks, supplemented with 10 weeks of combined NRT (patch plus choice of gum or lozenge). Participants in the LT-NRT arm (n=199) will receive 7 sessions of counseling extended over an entire year, focused on medication adherence and smoking reduction plus 52 weeks of combined NRT. Outcomes will be measured at 3, 6, and 12 months post randomization and will assess rates of smoking cessation, number of cigarettes smoked, tobacco smoke exposure, and clinical outcomes. In addition to comparing the effectiveness of treatment, a classification and regression tree analysis will allow us to identify subgroups of patients most likely to benefit from treatment.

Research team

Our research team is well prepared to conduct the proposed research and disseminate the research findings.

Edward F. Ellerbeck, MD, MPH, Principal-Investigator, has over 20 years of experience in measuring and improving the quality of medical care with 15 years focused on improving smoking cessation in underserved communities. He has directed focus groups with smokers,[52] directly observed smoking cessation practices in primary care settings,[53,54] and directed the first test of a 'chronic disease management' approach to smoking cessation in rural primary care practices.[55,56] He is currently directing a controlled clinical trial designed to enhance coordination of smoking cessation treatment for patients discharged from rural hospitals.[57] He serves on the smoking cessation committee of the Tobacco Free Kansas Coalition and serves as the Medical Director of the "U Kan Quit" smoking cessation service at KUMC.[58]

Kimber P. Richter, PhD, MPH, Co-Investigator, conducts research that focuses on gathering smokers' perspectives on smoking cessation,[59-61] the epidemiology of smoking,[62-65] treating nicotine dependence,[55,58,63,65-68] and multi-component health interventions in clinical and community settings.[58,69-72] She is Clinical Director of "U Kan Quit" at KUMC.[58,73] In addition to her research, she serves as president of the Association for Medical Education and Research in Substance Abuse (AMERSA), an organization that could help to disseminate the findings of the proposed study.

Nicole Nollen, PhD, Co-Investigator, has spent the past 13 years studying the determinants of health and health behaviors among underserved populations, evaluating promising behavioral and pharmacotherapy treatments for nicotine addiction, and examining psychosocial and biological mechanisms underlying tobacco use and treatment outcomes. She has been a co-investigator on four NIH funded clinical trials examining the efficacy of cessation pharmacotherapy in combination with motivational interviewing or health education counseling for quitting.[59,74-76] She is the PI of a currently funded R01 to examine racial/ethnic differences in quitting smoking among African American and White smokers treated with varenicline (Chantix). Within her studies, Dr. Nollen has focused on facilitating adherence to smoking cessation treatments. She led a pilot study that used adherence support counseling to achieve high rates of biochemically-confirmed rates of adherence to Chantix (86% over 3 months) in African American smokers.[77] Dr. Nollen led a separate investigation on adherence to bupropion and counseling also among African American smokers.[78]

Milind Phadnis, PhD, Co-Investigator is a Research Assistant Professor in the Department of Biostatistics at KUMC. His interest in biostatistics was stimulated by his experience as a Certified Quality Control Engineer and a Certified Reliability Engineer in the manufacturing industry. Accordingly, he procured his PhD in biostatistics from the University of Alabama at Birmingham, where he focused his studies on non-parametric methods in survival analysis and Bayesian mixed models. Dr. Phadnis is currently supporting a number of NIH-funded grants addressing health services research and is providing biostatistical and design support in the development of randomized clinical trials. Although Dr. Phadnis is still early in his career, he is a rising star who will have the protected time needed to complete the extensive analyses needed for this study. His work on this project will be supported by mentorship from his department chair, Matthew Mayo, PhD, a recipient of the 'KUMC Excellence in Mentoring Award'. Dr. Mayo has extensive expertise in clinical trials, biomarkers, and classification and regression tree analyses. (See attached letter of support.)

Study site

This study will be conducted at the Kansas University Medical Center (KUMC) and will take advantage of the infrastructure of the UKanQuit smoking cessation program. KUMC is an ideal setting for the conduct of this study. KUMC boasts a research participant registry and an electronic medical record (EMR) with an I2B2 research interface that will facilitate recruitment of participants. The smokers seen at KUMC come from urban and rural locations throughout Kansas, but mostly from Kansas City, Missouri and Wyandotte County, Kansas. (Wyandotte County is a majority minority county that has the dubious distinction of being one of the most disadvantaged jurisdictions in Kansas.) Our study site will allow us to enroll a diverse population of smokers for this study, including African American and Latino smokers with COPD. The experienced researchers in the UKanQuit program have an outstanding track record for recruitment of high risk smokers into clinical studies, recently enrolling more than 1,000 hospitalized smokers into an NIH-funded study designed to enhance utilization of tobacco quit lines.

Participants

Recruitment. As part of the Frontiers Clinical and Translational Research program at KUMC, we recently established a research registry that now contains 303 smokers with COPD that have already agreed to be contacted for studies such as this one; this registry is increasing in size daily. We will also recruit from the more than 1,500 smokers with COPD seen each year in the KUMC clinics and hospital. Recruitment of these smokers is enhanced by the use of a real-time, required field in the electronic medical record that identifies smokers when they are first seen. An automated referral system sends a daily list of newly identified smokers to our team of tobacco treatment specialists who can then approach patients about participation in this study. We will also recruit from Swope Health Services, Inc., a Federally Qualified Health Center that has helped us recruit more than 1,500 African American smokers to 4 NIH-funded trials. In the unlikely event that we are

unable to recruit sufficient patients quickly enough at KUMC and Swope, we will extend recruitment to the Midwest Cancer Alliance – hospitals affiliated with the KU Cancer Center, that have helped us recruit almost 1,000 smokers for two other NIH-funded smoking cessation studies, other local pulmonary practices or community venues providing services to smokers.

Eligibility. Patients will be eligible for enrollment in this study if they are 18 years of age or older, have physician-diagnosed COPD, smoke 5 or more cigarettes per day, have smoked 25 of the past 30 days (not including days spent in the hospital), and speak either English or Spanish. (Table 1) Patients will be eligible regardless of their interest in quitting as long as they are willing to take NRT. Patients will be excluded if they reside in a facility that doesn't allow smoking (e.g. certain nursing homes), lack access to a telephone, are suffering from a terminal illness with a less than 12 month life expectancy, or have an unstable cardiac condition. (NRT has been shown to be safe in patients with stable coronary artery disease,[45,46] but safety has not been adequately assessed in patients with unstable heart disease.) All patients must be willing to take NRT for up to 12 months, and be willing to complete 4 follow-up telephone-based counseling sessions and 3 follow-up visits. Although no formal assessment of psychiatric illness will be performed, subjects demonstrating markedly inappropriate affect or behavior will be excluded from the study. Everyone who attends a baseline session who is not eligible for the study but interested in quitting will be given self-help materials, such as *Clearing the Air*,[79] and will be referred to the state tobacco quitline. They will also receive a $20 ClinCard to compensate for their time and travel costs to attend the baseline visit.

Table 1. Summary of inclusion and exclusion criteria

Inclusion Criteria	Exclusion Criteria
• Aged 18 years or older<br>• Physician-diagnosed COPD<br>• Smoke ≥ 5 cigarettes/day<br>• Smoke cigarettes on ≥25 of the last 30 days<br>• Speak either English or Spanish<br>• Willing to take NRT for up to 1 year and participate in study procedures	• Reside in a facility that doesn't allow smoking<br>• Don't have an address and telephone<br>• Unstable cardiac condition (e.g. unstable angina or AMI in past 30 days)<br>• Pregnant or breast feeding<br>• Terminal illness with less than 12 month life expectancy

Randomization

All eligible participants who consent to participate in the study and complete the baseline data collection will be randomized to either SSC or LT-NRT. Randomization will be determined by computer-generated random numbers and a randomization module embedded in the data capture program.

Intervention

Both intervention arms will receive written smoking cessation materials and a series of 4 telephone-based counseling sessions and 1-3 in-person counseling sessions. Although the number, timing, and content of the counseling sessions will differ across the treatment arms, we anticipate that the total counseling contact time will be comparable, with one caveat. Participants in the SSC arm who are not interested in quitting within 30 days at the time of their baseline counseling session will not be offered additional counseling, which is consistent with most state quitlines. These participants will be informed that they may contact the program's hotline if they change their mind, at which point, counseling may either be reinstated or they will be referred to the state quit line for counseling. Participants in the SSC arm will receive 10 weeks of NRT contingent upon setting a quit date while participants in the LT-NRT arm will receive 52 weeks of NRT regardless of their immediate interest in quitting. An overview of the interventions is shown in Table 2.

Intervention-Smoking Cessation (SSC) arm

Written materials-SSC. SSC participants will receive written materials designed primarily to support the counseling sessions and provide the patient with a written documentation of their treatment plan. These materials will provide information on, benefits of quitting smoking, the importance of home smoking restrictions, and strategies for a successful quit plan, including information on relapse prevention. The content of the materials are consistent with those provided to smokers that call a tobacco quitline, but have been adapted to provide more information on NRT, including information on what to do with their NRT if they are still smoking, guidance on use of combination NRT (patch plus gum or lozenge), and troubleshooting suggestions if they experience adverse symptoms from the NRT. The materials will provide a number that the participants can call 24/7 if they believe they are having problems related to their treatment. Written materials will be in their language of choice (Spanish/ English). Due to varied literacy levels, materials will be reviewed with participants by study counseling staff. (Note: Materials were revised after convening our Stakeholder Advisory Committee and our Patient Advisory Panel.)

Table 2. Comparison of intervention in SSC and LT-NRT groups

	SSC	LT-NRT
Written Materials		
Cessation support	+	+
Reduction tips/replacing cigarettes with gum	+	+
NRT use and troubleshooting	+	+
NRT adherence	+	+
Counseling		
Counseling centered around quit date (weeks 0,1,3,6,10)	+	
Counseling on using NRT to cut back (weeks 0, 1, 3 & 6; Months 3, 6 and 9)		+
NRT Guidance and troubleshooting	+	+
Quit date and quit plan	++	+
Adherence support counseling	+	++
Reduction in cigarette support		++
NRT		
Combination therapy with patch & gum or lozenge	+	+
12 weeks of therapy, contingent on quit date	+	
52 weeks of therapy, all participants		+
+ indicates that the topic is addressed<br>++ indicates that the topic is covered in more detail		

Counseling-SSC. To maximize the generalizability of the study findings, the counseling sessions for the SSC arm will emulate services provided in a typical smoking cessation program such as a state tobacco quitline. The timing, duration, and content of each counseling session will be consistent with guideline-based recommendations,[13] and will be provided in the participants preferred language (English or Spanish). Dr. Richter will be responsible for the supervision and training on the SSC counseling protocol. Dr. Richter has extensive experience in development, training, and quality control of smoking cessation interventions, including quality monitoring for a nationwide quitline provider. Periodically, counselors will ask participants for verbal permission to audiorecord counseling sessions to demonstrate fidelity to study counseling protocols. Participants in the SSC arm will be offered up to 5 proactive counseling sessions with an in-person session at baseline and additional telephone-based sessions at weeks, 1, 3, 6 and 10 for those interested in quitting within 30 days. All counseling sessions will be delivered by a trained Tobacco Treatment Specialist.[80] Consistent with guideline recommendations, sessions will provide basic information about smoking and successful quitting. Participants ready to set a quit date will receive practical expert support in developing a personalized plan for cessation and long-term abstinence. They will learn to recognize danger situations, develop problem-solving and coping skills, and secure social support. Counselors will encourage participants in the quit attempt, communicate caring and concern, and encourage participants to talk about the quitting process.[13] Participants willing to set a quit date will also receive guidance on use of NRT, as outlined below. For SSC participants unwilling to make a quit attempt, the counselor will use a participant-centered approach to enhance the participant's motivation to quit as recommended in current practice guidelines.[13] They will be informed to contact the study hotline at any time during the study, at which point they will either be referred to the state quitline or provided counseling according to the SSC protocol if still within the first 6 weeks of baseline. The participant's current tobacco-use status will guide the content of each follow-up call. If the participant is quit, the counselor will review high-risk situations, coping skills, and stress management to prevent relapse. If the participant is still smoking or has slipped, the counselor will query circumstances related to the slip and troubleshoot avoiding these situations in the future. The counselor will then use cognitive behavioral techniques to build coping skills, with the goal of helping the participant build and implement a new

quit plan. The focus will then shift to increasing motivation to make another quit attempt, including confidence building, medication use, and cessation planning. The initial face-to-face session will last approximately 30 minutes and follow-up sessions will last on average 15 minutes. Rigorous attempts will be made to reach participants for all scheduled sessions with a minimum of 3 call attempts for each scheduled session including attempts during daytime and evening hours and on alternate days.

NRT-SC. Consistent with the current standard of care for smoking cessation, provision of NRT will be contingent upon the participant agreeing to set a quit date.[13] If they can identify a quit date, they will receive a 10 week supply of NRT and will be instructed to start the NRT on their chosen quit date. The dose of NRT will be adjusted to the participants smoking history as outlined in Table 3. The dose of the NRT will follow conventional treatment guidelines[13] and is consistent with the combined NRT treatment regimens that have been shown to be effective and safe in clinical trials.[81,82] Participants using NRT will be provided with both verbal and written instructions on how to use the NRT and how to avoid and manage any potential side effects. Participants will be instructed to use the nicotine patch on a daily basis even if they slip and have a cigarette. Participants will be asked to anticipate times that they typically smoke and use the gum or lozenge before deciding if they should have a cigarette. Smokers will be asked to try to consume at least 6-12 doses of gum or lozenge each day depending on their current smoking history. At each counseling call, the counselor will conduct a telephone-based assessment of the quantity of NRT used and will use this information to try to help the participant maximize their adherence to NRT.

Table 3. Dosing guide for NRT

Smoking history	Dose of NRT
Patch	
< 10 cpd	14 mg patch
10-20 cpd	21 mg patch
21 – 39 cpd	35 mg (1-21 and 1-14 mg patch)
≥40 cpd	42 mg (2-21 mg patches)
Gum or lozenge	
When in use with combination therapy	2 mg or 4 mg gum or lozenge every 1 – 2 hours

Intervention – Long-term Guided Maintenance Therapy (LT-NRT) arm

Written materials-LT-NRT. LT-NRT participants will receive the same written materials as those provided to SSC participants. This will include information on the benefits of quitting smoking and the importance of home smoking restrictions. Although the materials are identical, the smoking plan page that will be utilized in counseling and the instructions for completing it will be tailored to fit the arm, with participants in the LT-NRT group being instructed to start NRT immediately and continue its use longer term. They will receive the same information on use of combination NRT (patch plus gum or lozenge) and troubleshooting suggestions if they experience adverse symptoms from the NRT. Tips on medication adherence will be modified to address the long term nature of the treatment. As with the SSC arm, the materials will be available in either English or Spanish and will provide a number that the participants can call 24/7 if they believe they are having problems related to their treatment.

Long-term Guided Maintenance Therapy (LT-NRT). For the LT-NRT recipients, the counseling and NRT are integrally linked. While standard smoking cessation approaches (including our SSC comparison) address smoking cessation as a 'one-time event', our LT-NRT approach addresses smoking as a chronic disease that requires long term treatment. Indeed, our LT-NRT approach borrows heavily from the chronic care model of disease management[83,84] and mimics techniques of patient activation and medication management used in treatment of other chronic illnesses such as diabetes and hypertension. Accordingly, our LT-NRT empowers patients with the skills to manage their NRT, reduce their cigarette consumption, and prepare themselves for quit attempts as they become ready.

Each participant in the LT-NRT arm will receive 7 counseling sessions, In contrast to the SSC group, the timing of counseling sessions will be extended to support long term adherence to NRT. Counseling sessions will occur at baseline, and weeks 1, 3, 6 and Months 3, 6 and 9. The baseline and Month 3 and 6 sessions will be face-to-face and the others will be by phone. The initial face-to-face session will last approximately 30 minutes and follow-up sessions will last on average 10-15 minutes. Rigorous attempts will be made to reach participants for all scheduled calls with a minimum of 3 call attempts for each scheduled call, including attempts during daytime and evening hours and on alternate days. Counseling will be provided in the participants preferred language (English or Spanish). Dr. Nollen will be responsible for the supervision and training on the LT-NRT counseling protocol. Dr. Nollen has extensive experience in development, training, and

quality control of smoking cessation interventions and has adapted interventions to specifically support medication adherence in tobacco treatment. Supervision will include the periodic review of audiotaped counseling sessions to assure counselors are adhering to protocol components.

All participants in the LT-NRT arm will be asked to begin their NRT immediately after the baseline visit using the same dosing schedule outlined in Table 3. They will receive a 3-month supply of NRT at baseline and an additional 3-month supply after the Month 3, 6 and 9 counseling sessions. (*The timing of the resupply of medications mimics the 3 month medication supply typically used in healthcare settings for other chronic diseases – thus enhancing the potential for generalizability and adoption of the proposed intervention.*) The baseline visit will focus on the use of NRT, NRT adherence, and how NRT can be used to support smoking reduction. Smokers interested in making a quit attempt will be given cessation advice and supportive counseling as described in the written support materials. LT-NRT participants who continue to smoke will be asked to continue their NRT regardless of their current level of smoking and will be given supportive advice on maximizing adherence with NRT. LT-NRT participants who have quit smoking will be asked to continue NRT for a minimum of 6 months after they no longer experience smoking-related urges. If they have been abstinent for 6 months and no longer have urges to smoke, they will be provided a regimen for tapering their NRT dose with strict instructions for resuming the full dose if they have a slip or if smoking-related urges recur. LT-NRT participants will also receive adherence support counseling. Since some of the LT-NRT participants will not be ready for extensive counseling on quitting, the adherence support counseling will be particularly important. Adherence support counseling has been previously tested by our group[77] and will assist participants in identifying problems remembering to take their medication and help them develop strategies for remembering (e.g., timing doses with routine activities of daily living, posting reminders, setting alarms). Adherence support counseling will reinforce the continued use of NRT as one of the single best strategies for quitting or reducing smoking. This counseling will follow a semi-structured format that assesses smoking status and medication use, probes for difficulties participants are having with their medication, assists participants in problem-solving around issues they are having remembering to take their medications, and offers suggestions for dealing with self-reported side effects. Notably this counseling approach has resulted in high rates of adherence to smoking cessation medication (86%, biochemically verified). In the present study, we will not be able to biochemically verify adherence to NRT since nicotine in the blood could reflect either adherence to the treatment or continued smoking. Instead, adherence will be assessed using a 3-day recall of medication use. This approach is considered the gold standard when biochemical verification of adherence is not possible and in our own work has been validated with high sensitivity and specificity against medication blood levels.[77]

***Safety monitoring**.*

As with prior studies on combined NRT and use of NRT in continuing smokers, we anticipate few side effects with the treatment; however, as noted above, participants in both treatment arms will be prompted to report side effects at each contact and will be given the toll-free study phone number to report any adverse events at interim time points. Issues needing medical attention will be referred to Dr. Ellerbeck. We will follow NIH guidelines for reporting adverse events to the Human Subjects Committee, and address any unforeseen problems with our Data and Safety Monitoring Board. The DSMB will also receive quarterly reports to assure that side effects are not occurring at an unusually high rate.

In addition, if participants consent, we will inform their physician about their patient's participation in the study, providing them with basic details on the study along with our contact information so that they can reach us if they have questions or concerns.

Study visits and data collection methods

Trained research assistants will conduct assessments at baseline and 3, 6, and 12 months post-enrollment. Participants will receive a $50 ClinCard for each assessment completed, which is similar to a prepaid credit card and can be used almost everywhere that credit cards are accepted. Compensation will not be contingent on smoking status. To help remove transportation barriers for participants without transportation who live within a reasonable distance from one of our site locations, we will offer to provide cab vouchers for participants to get to and from their appointments. In order to offset this cost, participants must be willing to have their payment for that visit reduced to $30. To maximize retention, participants will be asked at the

baseline visit to provide contact information to facilitate follow-up: address, email, two telephone numbers if possible, and information for an alternate contact person. Reminder post cards and multiple calls will be used maximize attendance at follow-up visits. To facilitate follow-up, staff will keep flexible hours providing opportunities for follow-up during evening and weekend hours. Follow-up will be conducted primarily at one of our two Clinical Research Units – both of which have easy parking within 100 feet of the door. To further facilitate follow-up, research assistants will also try to time follow-up assessments so that they can be coordinated with medical visits at KUMC.

Measures

Outcome measures. Our selection of outcomes was derived from scientific expert consensus[85,86] and informed by input from our stakeholders and smokers with COPD. (Table 4) The primary outcome is biochemically verified 7-day point prevalence smoking abstinence defined as no cigarettes (not even a puff) for the previous 7 days at month 12. Biochemical validation will be based on expired CO, consistent with consensus statements from the Society for Research on Nicotine and Tobacco (SRNT).[86] For those participants who have reported they have quit smoking and who are unable to be verified in this way, we will obtain verbal permission and contact information for a household member or other regular contact to provide proxy validation. [104]

Table 4. Key outcome measures

**Construct**	**Measure** (assessment timepoint)
Smoking outcomes	
Smoking abstinence at 12 months*	7-day point prevalence abstinence, confirmed by exhaled CO or proxy validation (Mo 12)
6 month sustained smoking abstinence	Self reported 6 month abstinence at 12 month confirmed by exhaled CO or proxy validation (Mo 6 &12)
Quit attempts	Cumulative number of self-reported quit attempts lasting at least 24 hours (baseline, Mo 3, 6 & 12)
Average CPD	Average number of CPD (baseline, Mo 3, 6 & 12)
Smoke exposure	
Cumulative CO exposure over 12 months	Expired CO will be measured at each follow-up visit allowing an assessment of cumulative CO exposure. (baseline, Mo 3, 6 & 12)
NNAL exposure over 12 months	Urinary NNAL will be measured at each follow-up visit allowing an assessment of cumulative NNAL exposure. (baseline, Mo 3, 6 & 12)
Clinical outcomes	
Respiratory function	Simple spirometry will be performed at baseline and at the final follow-up visit. (baseline, Mo12)
Respiratory symptoms	Respiratrory symptoms will be measured using the COPD Assessment Test (baseline, Mo 3, 6 & 12)
Respiratory-related hospitalizations and ED visits	Intercurrent hospitalizations and emergency department visits will be assessed at each follow-up visit. Patients will be asked to classify the event as related to their heart or breathing. These will be validated by blinded physician review of medical records.
Cardiac-related hospitalizations and ED visits	

*Primary outcome

Secondary outcomes are 6 month sustained abstinence (biochemically verified), number of quit attempts, average CPD over the year of the study, and cumulative exposure to CO and NNAL. We will examine relapse rates 3 months after the conclusion of the study for participants who report they have quit smoking at the 12-month assessment. Clinical outcomes include both objective and subjective measures of respiratory function based on the measured FEV1 (as a percent of predicted) and the COPD Assessment Test.[87]

We will also track both respiratory and cardiac clinical events. This will be done by self-report of intercurrent emergency department visits and hospitalizations at each follow-up visit. We will validate both the occurrence and the nature of these self-reported clinical events by blinded physician review of medical records.

Although our primary outcome, biochemically verified smoking cessation is a standard outcome for smoking cessation studies and has been endorsed by scientific experts;[85] other outcomes included in this study have been driven by our patients and stakeholders. Our patients not only want to quit smoking, but want to feel better and spend less time in the hospital. They also would like to see that they are reducing their exposure to cancer-causing chemicals. Our stakeholders are very interested in the impact of the intervention on cessation and clinical events. These additional outcomes make this study relatively unique among smoking cessation studies. These additional outcomes are well aligned with our choice of focusing on participants with COPD. To date no controlled clinical trials have tested long term NRT in patients with COPD. Nevertheless, by focusing on patients with COPD, we have a much better chance of detecting meaningful differences in clinical outcomes associated with the intervention. Our study will also employ objective markers of smoke exposure (CO and NNAL). With rare exceptions, prior studies of long term NRT that looked at reduction in smoking used

self-report of CPD or they looked at nicotine metabolites (i.e. cotinine).[16,17] Self-report of CPD is highly problematic, because even if smokers really do reduce the number of cigarettes they smoker, they tend to smoke those cigarettes more efficiently,[16] and cotinine measurements are confounded by the use of therapeutic NRT. Our measures of CO and NNAL will allow us to have objective measure of actual smoke exposure. The NNAL is particularly relevant to our patients who would like to reduce exposure to carcinogens. NNAL (4-(methylnitrosamino)-1-(3)pyridyl-1-butanol) is a pulmonary carcinogen and metabolite of 4-(methylnitrosamino)-1-(3)pyridyl-1-butanone (NNK), a tobacco-specific nitrosamine related to the development of lung cancer.[88,89] NNAL assessed in urine is highly correlated with smoke-related tobacco exposure.[90]

Covariates

We will collect a variety of covariates (Table 5). These covariates will allow us to verify the comparability of the two comparison arms at baseline and will also allow us to estimate treatment effects for given personal characteristics (see discussion of CART analysis in the analytic plan). We will assess demographic variables, social history variables that might influence tobacco consumption, smoking related variables, including cpd, nicotine dependence, and biological markers of nicotine consumption, and clinical variables including both objective and subjective assessment of respiratory function and prior history of respiratory-related clinical events.

Table 5. Co-variate measures

**Construct**	**Measure** (assessment timepoint)
Demographics	gender, age, race, ethnicity, income, education, employment, marital status, family structure (baseline)
Social history	Other smokers in the home, presence of home smoking restrictions, anxiety (GAD-2), depression (PHQ-2), health history (baseline)
Smoking Variables	
Smoking history	Quantity smoked (baseline, Mo 3, 6 & 12), quitting history, use of other tobacco and nicotine products, including e-cigs and NRT, (baseline)
Readiness to quit	Interest in quitting will be be assessed using 30 day anchor (stage of change) and 12 months (intent to cut back or quit) (baseline, Mo 3, 6 and 12)
Nicotine dependence	*Fagerström Test for Nicotine Dependence (FTND),*[91] time to first cigarette (baseline, Mo 12); Brief WISDM[105] among continued smokers at month 12.
Nicotine intake	Urinary cotinine/creatinine ratio (baseline, Mo 3, 6, and 12)
Clinical Variables	
Respiratory function	FEV1 (baseline)
COPD symptoms	CAT score, # of hospitalizations for COPD in past year (baseline)
Treatment Variables	
Medication adherence	3-day medication use recall (Mo 3, 6, and 12)
Counseling adherence	total counseling sessions completed

Analytical Plan.

Sample Size and Power Calculations. Our sample size was based on our primary outcome, point-prevalence abstinence at Month 12, using an intent-to-treat approach. Based on prior studies using precessation NRT, we estimate a 2-fold increase in cessation outcomes in the LT-NRT group compared to the SSC group.[17-19,26] Given our recruitment of patients at all levels of readiness to quit, we estimate a 12 month cessation rate of 10%.[56] With 199 participants, in each arm, we will have an 80% power to detect a 2-fold difference or greater with a Type I error rate of 5%. This sample size will provide comparable power for looking at 6 month sustained cessation. For the CO measured longitudinally at four time points, we have 80% power to detect a mean difference of 2.24 ppm. Likewise, for NNAL, we have 80% power to detect a mean difference of 0.41 pmol/mg creatinine or more between the two groups.

Specific Aim 1: Compare the benefits of traditional smoking cessation (SSC) versus long-term guided maintenance therapy (LT-NRT) with NRT. The chi-square test will be used to compare the verified 7-day point prevalence abstinence at Month 12 between the two groups. If there is imbalance between treatment groups at baseline, we will switch to logistic regression for our primary analysis. We will use similar methods to test for group differences for dichotomous secondary outcomes including sustained abstinence and proportion with 1 or more ED visits or hospitalizations. To test for differences in change in respiratory symptoms and FEV1 from baseline measured at 3, 6, and 12 months, we will use standard mixed models repeated measures analysis [92] Likewise, to test for differences in longitudinal exposure to CO and NNAL, we will compare the area under the curve over the entire 12 months of follow-up using repeated measures analysis. If the treatment differences are heterogeneous across any covariate sub-groups, we will quantify and report such interactions

with appropriate contrast statements. We will compare the cumulative number of self-reported quit attempts by means of a Wilcoxon rank-sum test.

As smoking cessation trials are likely to have missing data, we will adopt the approach of Hedeker et al.[93] for analyzing the primary binary outcome of smoking abstinence. We will initially conduct our analyses ignoring missing data and then conduct sensitivity analysis using three different approaches. The first approach will impute data under the two extreme assumptions of ‘missing = smoking’ and ‘missing = non-smoking’. The second approach will impute data using the last observation carried forward method, and finally we will conduct multiple imputations for missing data, modeling the uncertainty inherent in individual patient outcomes using a logistic regression model.

Specific Aim 2: **Develop estimates of patient-desired outcomes based on patient-specific characteristics and treatment choice (SSC vs. LT-NRT)** We will employ a Classification and Regression Tree (CART) analysis to identify subgroups of participants whose personal characteristics might impact the benefit of treatment. The resulting decision tree will allow smokers and their care providers to estimate their individual chances of success given their choice of treatment. To maximize the utility of the decision tree, we will start by soliciting input from our Stakeholder Advisory Committee and Patient Advisory Panel. We will ask them to identify key demographic, psychosocial, biological, and smoking factors from Table 5 and help us convert them into clinically meaningful binary variables that could be tested for inclusion in a clinically meaningful decision tree. (Drs Ellerbeck and Phadnis will provide both groups with simple conceptual models to assist them in providing meaningful, independent contributions.) The candidate variables, along with treatment assignment (LT-NRT vs SC) will be entered into a recursive partitioning model as described by Brieman and Freidman using the RPART package in the R statistical software.[94] Specific methods related to building the tree (using appropriate splitting criteria and loss incorporation using the altered priors method), pruning the tree and handling missing data (using surrogate variables) will be deployed using the techniques described by Therneau et al.[95] Given the event rates expected and overall sample size, a validation subsampling scheme will not be feasible, thus we will utilize 10-fold cross-validation on the regression tree to assess its properties. Additionally, we will account for the uncertainty in tree classification by generating a bootstrap sampling distribution of the CART as described by Shalizi.[96] In addition to developing a CART model/decision tree for smoking cessation, separate classification models will be developed for other high priority binary outcomes, including: 1) 6 month sustained abstinence; 2) 50% reduction in carcinogen (NNAL) exposure; and 3) COPD related hospitalization (yes/no). (These were the models of greatest interest for our initial Patient Advisory Panel, but other outcomes could be modeled as well if our patients and stakeholders think that these additional models might be useful.)

Demonstrate the patient-centeredness of the application (*Criterion 4*)

By comparing the effectiveness and safety of two alternative approaches valued by patients to reduce harm from cigarette consumption, our research proposal fits squarely in the priority area: ‘Assessment of Prevention, Diagnosis, and Treatment Options’. It matches closely with 3 of the top 10 PCORI Comparative Effectiveness Research Priority areas: Prevention (preventing harm from smoking related diseases, e.g. lung cancer), Chronic Disease (reducing smoking related complications from chronic diseases (e.g. COPD), and Patient Engagement (building upon risk reduction approaches currently being used by many patients, but not widely promoted in the health care community). Our analytic methods will provide specific answers for patients with key PCORI-like questions such as:

- ‘Given my personal characteristics and preferences, what should I expect will happen to me if I try to quit right away versus start long term nicotine replacement?’;
- ‘Is long-term nicotine replacement a realistic option for me?’;
- ‘What are the potential benefits and harms of long-term nicotine replacement versus trying to quit right away?’; and
- ‘What can I do to improve the outcomes that are most important to me, such as reducing my smoking, reducing my exposure to cancer-causing toxins, and ultimately being able to quit smoking long term?’

Our research specifically addresses current gaps in knowledge related to the role of long-term NRT in reducing harms of cigarette smoke. The findings have the potential to influence decision-making of key stakeholders related to smoking cessation guidelines and reimbursement for smoking cessation treatment. Our study is inclusive of different populations in both the planning and implementation of the research, by including low-income smokers, African Americans, and Latinos. Finally, the development and implementation of the study are highly patient centered, addressing research questions posed by smokers and outcomes valued by smokers with COPD.[97]

We utilized a variety of approaches to ensure the engagement of patient's and stakeholders in the design and conduct of the proposed research project. We reviewed the literature to identify patient-centered research studies that captured the perceived needs and interests of smokers and patients with COPD. We reviewed guidelines and consensus statements in order to capture the perceptions of key stakeholders. We spoke directly with smokers with COPD that have been grappling with issues related to smoking cessation and harm reduction. We conducted semi-structured interviews with these smokers, soliciting their perceptions on critical components related to the design and conduct of the proposed study. We also convened a Patient Advisory Panel that has provided critical input into this proposal and will continue to guide the investigators throughout the conduct of the study. Finally, we used the support of the Tobacco Free Kansas Coalition to bring together key stakeholders, including health care providers, public health experts, and representatives of key stakeholder organizations. Each of these approaches is delineated below.

Our research addresses issues raised by patients and stakeholders as illustrated by a national survey of 1,000 smokers.[15] Of these smokers, 29% had no interest in changing their smoking habits over the ensuing year, but the other 71% varied substantially in how they wanted to address smoking cessation or harm reduction. While one in four were interested in the traditional approach to smoking cessation; the other 3/4[ths] wanted to either cut back on smoking but not quit completely (harm reduction) or, more commonly, gradually reduce their smoking as a prelude to quitting. The majority of these smokers were interested in using nicotine replacement to help them reduce the number of cigarettes that they smoke with slightly more preferring patch than gum. These findings were corroborated by the ITC Four Country Survey which showed that many smokers are already using NRT for reasons other than quitting.[98] In light of these findings, it is not surprising that current cessation programs are failing to reach the majority of smokers. Not surprisingly, the Consumer Demand Roundtable of the National Tobacco Cessation Collaborative concluded that current clinician-oriented cessation programs are only reaching a small portion of the patients in need. They concluded that the needs, interests and perspectives of smokers needed to be heard in order to develop more effective strategies.[99]

In recognition of this, we asked our patients to help us develop this research proposal, including the choice of the research question, the design of the intervention, and the outcomes included. We started by conducting structured interviews with 21 smokers with COPD. While only a minority of these smokers (7), were ready to try to quit within the next 30 days, almost all were interested in cutting down, primarily to improve their health, improve their breathing, and reduce their risk of cancer (outcomes that we have incorporated into this study). Some expressed concerns about the safety of Chantix. Most (18) were very interested in the idea of using NRT long term to either help them quit or simply to reduce their exposure to the harm from cigarettes, and the majority were interested in participating in a study that would help them reduce their smoking, even if they had to take medications long term.

We invited five smokers with COPD to serve on a 'Patient Advisory Panel' to tell us about the issues that were most relevant to them, to help us interpret the results of our literature review and structured interviews, and to guide us in choices related to research questions, study design, and relevant outcomes. The Panel reinforced the findings of the structured interviews related to important outcomes. They voiced strong support for an intervention that would allow them to quit gradually, and they were very interested in using NRT prior to quitting, using NRT instead of Zyban or Chantix, and using combination NRT. They warned us that very few patients will have tried combination therapy with NRT, so this will have to be addressed in our patient education, but they thought the idea of using combination therapy would be very appealing to many smokers. They also warned us of the need to create clear 'troubleshooting procedures' for the NRT and recommended that we provide choices of gum versus lozenge and offer different choices of flavors.

Although patient engagement was critical to the design of this study, we recognize that implementation of research findings will be critically dependent upon the support of other stakeholders, including health care providers, public health experts, and the community organizations that have been at the forefront of the fight against tobacco. Drs. Ellerbeck and Richter have worked extensively with the Tobacco Free Kansas Coalition (TFKC) – a coalition of community organizers, private voluntary organizations, health care providers, and public health representatives. Drs. Ellerbeck and Richter continue to serve on the cessation committee of the TFKC, and Dr. Richter currently serves on the board of directors. On behalf of the TFKC, Drs. Ellerbeck and Richter personally testified at local and state-level government hearings across Kansas in support of clean indoor air laws; they continue to support the organization with scientific guidance and testimony on tobacco related issues. In support of this project, the TFKC helped the researchers identify stakeholders to serve on the Stakeholder Advisory Committee who, in turn, are advising us on the design and conduct of this study. This committee includes Linda DeCoursey, Executive Director, TFKC; Jim Gardner, MD, primary care physician and TFKC board member; Jon Hauxwell, MD, primary care physician and former TFKC board member; Matthew Shrock, Kansas state quitline coordinator, Joan Smith, public health practitioner and advocate; John Neuberger, PhD; epidemiologist/toxicology expert; Deb Parsons, American Cancer Society; Kevin Walker, American Heart Association; and Beth Marolf, American Lung Association. (see attached letters of support) This subcommittee reviewed this proposal and provided critical input into the study design, including issues related to participant inclusion, outcome measurements, and treatment. The health care professionals on this committee shared with us their frustration with the current treatment options for patients with COPD that smoke; they were particularly enthusiastic about the potential of initiating long term NRT to reduce smoke exposure and enhance the potential of ultimately quitting.

Demonstrate the commitment to patient and stakeholder engagement (*Criterion 5*)

Prior history of patient and stakeholder engagement. Dr. Ellerbeck is a practicing physician who engages with smokers and patients with COPD on a regular basis in his clinical practice. He and his team of co-investigators are deeply indebted to the health care providers and smokers throughout Kansas that have played a critical role in their past work. He and Dr. Richter visited more than 50 clinics and hospitals around the state of Kansas to discuss smoking cessation and identify better ways to integrate smoking cessation into clinical practice. The insights gained from this work were critical to the successful implementation of the UKanQuit program at KUMC.[58,73] The physicians in the Kansas Physicians Engaged in Prevention Research practice-based research network helped with the design and implementation of a chronic disease model for smoking cessation.[56] Smokers throughout rural Kansas gave us insights into the culture of rural smoking,[100] and showed us how this model could be adapted to address the needs of smokers in rural primary care.[56,67] Smokers in drug treatment shared their experiences on cessation attempts and their strong interest in harm reduction,[100] leading to a variety of efforts to try to improve tobacco control in the context of drug treatment.[101-103] We built upon the lessons learned in these past efforts to engage patients and stakeholders for this project.

Patient and Stakeholder engagement to date for this project:
As described in the previous section, we have already engaged patients and stakeholders in the design of this research project and the formulation of the research questions, conducting structured interviews with smokers, convening a Patient Advisory Panel and meeting with our Stakeholder Advisory Committee. The findings from this engagement are described under Criterion 4 and incorporated throughout the actual design of the study (Criterion 3).

Plan for ongoing patient and stakeholder engagement:
At the heart of our ongoing engagement plan is our Patient Advisory Panel. We have already engaged a preliminary group of panel members, but will recruit additional panel members to enhance representation of our multicultural patient population. Our Patient Advisory Panel will include patients with COPD, some who have quit and some who continue to smoke. Our final panel will consist of approximately 8 members representing the full diversity of our COPD population. We will engage our stakeholders and local health care providers to help us identify potential panel participants. We will convene our panel members immediately

upon notice of award so that they can review the study design, recruitment methods, interventions, and outcomes assessments. Meetings will be conducted at convenient times with free parking and easy access. (Some panel members will be using oxygen.) We will provide a $75 stipend to cover their time and the cost of transportation for each meeting. Panel members will convene semi-annually, but our initial panel expressed a willingness to use telephone and e-mail on an as-needed basis between meetings. We will do our best to incorporate input from Panel members into the study so long as it does not compromise the technical merit of the research design. We plan to hold one meeting each year in conjunction with the Stakeholder Advisory Committee (see below). At each meeting panel members will receive project updates and we will solicit their input on project-related activities such as participant recruitment, data analysis, and interpretation of study findings. Meetings of the Patient Advisory Panel will be led by Tresza Hutcheson, a psychologist with substantial experience in engaging participants in open discussion.

Our Stakeholder Advisory Committee (described under Criterion 4) will continue to work on the planning and implementation of this study. The Committee convened immediately upon notice of study award to provide input into any modifications needed in the study design, recruitment methods, interventions, or outcome assessments. This initial meeting was particularly important in verifying that our study can lead to results that would be actionable by the stakeholders. Committee members will reconvene twice a year, once by conference call and once face-to-face, in conjunction with our Patient Advisory Panel. At these semi-annual meetings, committee members will advise the investigators on a variety of issues including participant recruitment, data analysis, publication, and dissemination of results. Committee members also expressed a willingness to meet by phone or e-mail on an as-needed basis.

Taking advantage of patients and stakeholders to help disseminate actionable findings

Both our Patient Advisory Panel and our Stakeholders will be highly engaged in our analysis plans and dissemination of results. We will work with both Panel members and Stakeholders to identify opportunities for joint presentations, publications, or media events. Panel members, in particular, will be important in formulating press releases and speaking to the media. Both Panel members and Stakeholders will be prepared for these presentations and events since they will have been engaged in planning the analysis (particularly the CART model and decision tree). Prior to publication and dissemination of findings, the Stakeholder Advisory Committee will review scientific reports and drafts of publications to ensure that the messages included in these reports are relevant to clinicians and policy makers. In year 3 of the project, we will have a combined meeting of the Patient Advisory Panel and the Stakeholder Advisory Committee to review findings in lay language, assuring that the dissemination of the findings reflects the underlying scientific findings, but can also be easily understood by the community at large. Both groups will help us incorporate findings into our website and disseminate findings to the public (e.g. press releases). Furthermore, panel members will help design our study website. In addition to highlighting study findings and protocols, the website will allow interested patient advisors or study participants to share personal stories of their experiences of quitting smoking, smoking with COPD, etc. We will also work closely with our Stakeholder Advisory Committee to get study findings into the hands of guideline developers and key national stakeholders.

Working jointly with our Patient Advisory Panel and our Stakeholder Advisory Committee, we will take advantage of our connections with national organizations and smoking cessation experts to disseminate results. As president of AMERSA (Association for Medical Education and Research on Substance Abuse), Dr. Richter has ready access to some of the nation’s leading experts on nicotine addiction and smoking cessation. We will take advantage of listserves established by AMERSA and SRNT (the Society for Research on Nicotine and Tobacco) to discuss findings with other researchers. We will also use meetings of the Society for Research on Nicotine and Tobacco, the Society for Behavioral Medicine, and the Society for General Internal Medicine as opportunities for networking and dissemination.

DISSEMINATION AND IMPLEMENTATION POTENTIAL

Our study and analytic plan are well designed to produce results that are amenable to widespread dissemination and rapid implementation. Dissemination will occur through presentations at national meetings, publications in the peer-reviewed literature, distribution of study findings in the lay media, development of a study website, and adoption of study findings in clinical practice guidelines and by advocacy groups. To enhance implementation of the study findings, the patient-centered decision tree (Aim 2) will be posted on the web and can be incorporated into electronic health records. Perhaps the most critical aspect of implementation, however, will be the use of these study findings to advocate for policy changes in coverage of NRT by insurance plans.

We will subject study findings to peer review at national meetings and in the peer-reviewed literature. Drs. Ellerbeck, Richter, and Nollen plan to present the findings of this study at a variety of national meetings such as the Society for Research on Nicotine and Tobacco, the Society for Behavioral Medicine, and the Society of General Internal Medicine. Findings will also be published in the peer reviewed-literature. Publication is critical to procuring the attention of advocacy groups and guideline developers. Along with publication and presentation of our findings, we will take advantage of the KUMC media department to prepare press releases related to our study. We have a strong track record in procuring regional and national coverage of our research. (We plan to make extensive use of or Patient Advisory Panel and our Stakeholders in this part of the dissemination process.) We will also work with PCORI staff to support dissemination on their website, listserve, and other distribution channels.

Our Stakeholder Advisory Committee includes representatives of some of the most prominent national and regional advocacy groups concerned with smoking related illnesses. We will work with both the regional and national offices of these stakeholders to identify channels of distribution of the study findings and identify ways to get the study findings incorporated into their organizational guideline development programs.

The most prominent guideline related to tobacco control policy is the guideline produced by the US Preventive Services Taskforce.[13] Our investigators know many of the authors of these guidelines personally and interact with them at national meetings, in particular through the Society for Research on Nicotine and Tobacco. We will work with these guideline developers to see if the findings of our study can be incorporated into the next update of these guidelines. Incorporation of study findings into clinical practice guidelines can have tremendous implications for driving policy changes that can drive implementation of these findings.

Dissemination to patients, stakeholders (particularly clinicians), and policymakers, as outlined above, are critical to implementation, but implementation will also be facilitated by the publication of our ‘decision tree’ which will help patients and clinicians understand the relative merits of treatment choices given the smokers unique characteristics (Aim 2). This decision tree will be published on the web and made accessible world-wide. We propose to incorporate the decision tree into our own EPIC electronic health record at KUMC and share this adaptation to the EPIC learning community. EPIC is the most widely used electronic health record in the United States and EPIC users have a variety of tools available for sharing innovations across institutions, including an EPIC users group associated with the NIH’s Clinical and Translational Research Centers program of which KUMC is a member. As the decision tree gains acceptance, it can be distributed across other electronic health record platforms.

Perhaps the most important step in the implementation of study findings will be related to making changes in insurance coverage for NRT. Insurance coverage for NRT varies substantially across health plans, but in general, insurance companies have placed limits on the duration of NRT use and do not cover combination therapy. Dr. Ellerbeck has been directly involved in working with state Medicaid programs on revising coverage of services for smoking cessation. Given specific provisions within the affordable care act, making these changes in coverage decisions will be much easier if the findings of this study are incorporated into

clinical practice guidelines – as noted above.

Describe possible barriers to dissemination and implementation of your work in other settings.

There will be several potential barriers to dissemination and implementation of our study findings. Perhaps the biggest barrier will be the entrenched attitude in both the practitioner and lay community that smoking is a 'personal choice' and therefore smoking cessation services shouldn't be covered under insurance policies. Indeed, there has been a long history of insurance companies and employment benefit plans excluding treatment coverage for 'lifestyle choices' such as smoking cessation. We believe that new understanding of the neurophysiology of nicotine addiction is changing these attitudes, but the attitudes persist nonetheless.

The next biggest barrier will be getting the actual changes made in the guidelines. Guideline development takes time and requires negotiation among many individuals with varying interests and backgrounds. Our study, however, directly addresses many of the research priorities delineated in the most recent iteration of the guidelines and the technical rigor of our study design and analytic plan should facilitate incorporation of findings into future guidelines.

Finally, getting clinicians to actually incorporate research discoveries into clinical practice has long been a challenge. Indeed, our research team has already demonstrated the difficulties that many primary care practitioners have in incorporating existing guidelines into their practice.[53,58] We have, however, been at the forefront of efforts to integrate tobacco treatment services into electronic health records[73] and we believe that integration into electronic health records, along with performance monitoring could, at least in part, address this concern.

REPRODUCIBILITY AND TRANSPARENCY OF RESEARCH

While the findings generated from this proposed research have direct relevance to the 5 million Americans with COPD that smoke, this study could be easily replicated in other populations with implications for the 43 million smokers in the United States. Our protocol, intervention materials, and counseling procedures will be freely available to facilitate implementation of our study findings into clinical practice or replication of our study in other populations. Our study will include a diverse population of smokers representing low-income patients, whites, African Americans, and Latinos. Our CART analysis will allow us to test for differential effects based on ethnicity, but this analysis would not preclude efforts to replicate this study among Latino or African American smokers, who have been noted to have lower rates of smoking cessation than white smokers. The protocol could also be readily adapted for use in the American Indian population which has particularly high rates of smoking and low rates of success with traditional cessation efforts.

Findings could also be replicated in other clinical populations, including populations with other chronic diseases such as heart disease, diabetes, substance abuse disorders, or isolated nicotine dependence. Larger sample sizes, however, might be required to identify changes in clinical outcomes in these populations since rates of clinical events would be expected to be much lower.

Reproduction of Research Findings (Data Sharing Plan)

Note: The requirement for a data-sharing plan applies only to studies that are requesting funding at a level greater than $500,000 in direct costs in any project year. The data sharing plan must:

- *State that a complete, cleaned, de-identified copy of the final dataset used in conducting the final analyses will be made available within nine months of the end of the final year of funding.*
- *Propose a method by which investigators will make this dataset available if requested.*
- *Propose a budget that would cover costs of data sharing if requested.*

Although a formal data sharing plan is not required for this study, data sharing is an integral part of the many systematic reviews that support the recommendations of existing practice guidelines. We will therefore plan to create a clean, de-identified copy of our final dataset that will be available to other researchers, upon request, within 9 months of the end of the final year of funding. Prior to data sharing, we will remove or convert all identifying information (date of birth will be converted to age and other identifiers will be removed).

There may remain the possibility of deductive disclosure of subjects with unusual characteristics. Thus, we will make the data and associated documentation available to users under a data-sharing agreement that provides for: (1) a commitment to using the data only for research purposes and not to identify any individual participant; (2) a commitment to securing the data using appropriate computer technology; and (3) a commitment to destroying or returning the data after analyses are completed. Data will be saved as SAS or SPSS files, saved to disk, encrypted, and mailed to users. A copy of our analytic code used to generate our primary outcomes paper(s) will also be available on request. Given that this study does not meet the threshold for data sharing requirements, the costs of data preparation and shipping will be borne by the requestor, but since the data files will already be constructed, these costs will be nominal.

PROTECTION OF HUMAN SUBJECTS

Risks to the Subjects

Human Subjects Involvement and Characteristics
The primary research interest of this study is to compare the effectiveness of a standard smoking cessation program versus long-term guided maintenance therapy with nicotine replacement that would support reduction in the amount smoked and set the stage for later quitting. Patients will be eligible for enrollment in this study if they are 18 years of age or older (although smokers younger than 40 are unlikely to have COPD), have physician-diagnosed chronic obstructive pulmonary disease (COPD) (asthma and chronic bronchitis will be included), smoke 5 or more cigarettes per day, have smoked 25 of the past 30 days, and speak either English or Spanish. Patients will be eligible regardless of their interest in quitting. Patients will be excluded if they reside in a facility that doesn't allow smoking (e.g. certain nursing homes), lack access to a telephone, are suffering from a terminal illness with a less than 12 month life expectancy or have an unstable cardiac condition. All patients must be willing to take NRT for up to 12 months, be willing to complete 4 follow-up telephone-based counseling sessions, and be willing to complete 3 follow-up visits. Although no formal assessment of psychiatric illness will be performed, subjects demonstrating markedly inappropriate affect or behavior will be excluded from the study. Everyone who attends a baseline session who is not eligible for the study but interested in quitting will be given self-help materials such as *Clearing the Air*,[79] and will be referred to the state tobacco quitline.

The personnel for this application will complete HIPPA and Human Protection Tutorials Certification prior to starting this project. During the recruitment and informed consent phases, participants will learn about the study and will be informed that their participation is voluntary and that they can withdraw from the study at any time without impacting their current or future health care. Upon enrollment, all enrolled participants will have the highest level of protection of confidentiality for their participation in this study. The potential subject will be given ample opportunity to ask questions prior to agreeing to participate. This entire process will be observed by a witness and will be documented in writing. Dr. Ellerbeck will be available to answer questions and address any concerns throughout the study. He is available by pager or cell phone 24/7; on dates when he may be unavailable, he will arrange for appropriate study coverage. Dr. Ellerbeck will retain copies of the signed consent forms throughout the course of the study. Standard language in our consent form assures the participant of the confidential nature of our study. These standards are strictly adhered to and monitored by the University of Kansas Human Subjects Committee. Assigning each participant a study number, numerically coding all data, and secure file storage will maintain confidentiality. A key linking ID-codes and participant's names will be kept on a secure, password-protected server. Only summaries of findings will be reported in any publications or presentations, with no identification of individuals.

Sources of Materials
This study involves testing alternative methods for reducing the harms of smoking and supporting smoking cessation among smokers with COPD. We will provide participants with standardized information on smoking cessation and the appropriate use of NRT. We will gather data from all of our study participants at baseline and months 3, 6, and 12. Information gathered will include surveys, medical records information, measures of respiratory function, urinary measures of nicotine and cigarette smoke exposure, and measures of expired carbon monoxide (to measure smoke exposure). The baseline questionnaire will collect information related to demographics, smoking history, and health status questions. At baseline, 3, 6, and 12 months, we will collect urine specimens, exhaled CO and will administer a brief survey assessing their current smoking, respiratory symptoms, and history of COPD-related hospitalizations or emergency room visits. In addition, at baseline and Month 12, we will collect a measure of their breathing (i.e., spirometry).

All questionnaires and informed consent documents will be submitted to the KUMC IRB for approval prior to implementation.

The KUMC Department of Preventive Medicine and Public Health has a well-developed structure for data management. Working data is maintained on a single large file server. Inactive files are moved to archival storage under control of an automated system, itself controlled by a DBMS (Ingres) based request system that ensures that all data movement is appropriately logged and commented. The archival storage is hosted on the institutional mainframe computer, which also supports billing and registration. The use of the mainframe ensures several high level support functions for the archive system (e.g., storage in separate fire zones, regular copying of data to new media, and guaranteed availability).

The data management will be governed by standard procedures for data security and access. All analyses are logged with respect to IRB authorization, accounting information, principal and co-investigators, statistician, and data analyst involved in the analysis. In order to create a unified data management strategy, we will identify all subjects with a study_id. To ensure subject confidentiality, no names, social security numbers, hospital or clinic numbers will be included in the shared databases. Names, addresses, telephone numbers, and any other information needed for recruitment, study involvement, and tracking will be obtained and maintained locally by the project personnel. All computer files and systems will be password protected and accessible only by authorized personnel.

Potential Risks.
Risks for participating in the study are primarily those related to the use of NRT. The safety of NRT has been demonstrated in multiple studies, including studies of patients with a history of stable heart disease. To minimize any possible risk, we will exclude any patients with unstable heart disease. Nevetheless, NRT, particularly in continuing smokers, can be associated with nausea, jitteriness, insomnia, and vivid dreams.

Risks also include those associated with the inconvenience of participation including answering surveys and participating in follow-up visits and assessments. No data are collected that would put participants at risk for criminal or civil penalties.

Alternatives
Alternatives to participating in the study are to quit “cold turkey” (without assistance), use other smoking cessation programs, purchase other NRT from their pharmacy, obtain a prescription for varenicline, bupropion or other smoking cessation products from their physician, or continue to smoke.

Adequacy of Protection Against Risks
Recruitment and Informed Consent
We will recruit potential participants through our participant registry, through the UKanQuit smoking cessation program, and through direct mail to potential participants identified through the I2B2 interface to our electronic health record. (These mailings would come from collaborating physicians at KUMC.) Research Assistants, who have completed special training in human subjects protection, will contact smokers who have been referred by their doctors, referred by the UKanQuit program, or notified by their physician of the study and not declined contact. These Research Assistants will inform patients of the study, collect assent for doing the eligibility assessment, and then conduct that eligibility assessment. For eligible patients, additional contact information will be obtained and we will offer to mail a copy of the consent form to review prior to the first in-person visit. We will arrange a baseline visit at which time we will obtain written, informed consent from participants who are eligible and interested in participating.

Patients who are not eligible or do not wish to participate will be informed that they will still receive their usual care from their physician and will receive a referral for the state tobacco quitline. If they are excluded or decide not to participate at the baseline visit, they will be offered written smoking cessation materials. For those interested and eligible, the consent form will include a description of study procedures, the time involved, the right to withdraw at any time without penalty, procedures used to protect participant confidentiality, data collected in the study and the use of data, and potential benefits and risks of participating in the study. All informed consent documents will be submitted to, and approved by, the University of Kansas Medical Center’s Human Subjects Committees. Signed consent forms will be kept in a locked cabinet. During the recruitment phase, the potential participant will be informed of the details of the study and the fact that participation in this study is entirely voluntary and will not affect their current or future medical care at any medical facility. The

smoker must successfully complete eligibility screening and provide informed consent before being enrolled in the study. This ensures that all participants meet all inclusion/exclusion criteria as stated in the study protocol and in the informed consent provided to the participant.

Protection Against Risk

Risks for participating in the study are primarily those related to the use of NRT. The safety of NRT has been demonstrated in multiple studies, including studies of patients with a history of stable heart disease. To minimize any possible risk, we will exclude any patients with unstable heart disease. Nevertheless, NRT, particularly in continuing smokers, can be associated with nausea, jitteriness, insomnia, and vivid dreams. To address these concerns, participants will receive written information and information from their counselor about how to manage any of these symptoms. They will be given advice on when they might need to reduce their nicotine dose (whether through decreasing smoking or decreasing NRT). We will track adverse events and present these adverse events to our data safety and monitoring board (see data safety and monitoring plan below).

To minimize the inconveniences associated with study participation we have reviewed study procedures and data that will be collected with our Patient Advisory Panel and our Stakeholder Advisory Committee. We have used their input to minimize the number of items in our instruments and improve the accessibility and convenience of our study procedures. We anticipate using several methods to enhance convenience to participants, including offering study visits in the evening and on weekends and at our two clinical research units that include easy parking and access to mass transit. We will also try to coordinate assessments with physician visits.

To minimize risks related to confidentiality, we will maintain strict adherence to HIPAA standards and follow the data security procedures previously described. We will avoid collecting any data that could put participants at risk for criminal or civil penalties. Standard language in our consent procedure assures the participants of the confidential nature of the study. Those who elect to participate will be clearly told that they may withdraw from the study at any time without jeopardizing current or future care at any medical facility. Potential participants will also be informed of alternative treatments (i.e., using other smoking cessation programs, purchasing nicotine gum, patches, or lozenge from the pharmacy, obtaining a prescription for nicotine inhaler, nasal spray, or other smoking cessation products from their physician).

The consent form will be reviewed with each participant and they will be given a copy of the signed consent form. These standards are strictly adhered to and monitored by the KUMC Institutional Review Board. Only summaries of group data will be reported in any publications or presentations, with no identification of individuals. All records will be kept in locked filing cabinets in offices that are kept locked when unoccupied. Subject files will be kept in a secure area, with access only by designated researchers who are registered with the IRB as being affiliated with this study.

Potential Benefits of the Proposed Research to the Participants and Others

Quitting smoking is one of the best things a smoker can do for his or her health regardless of whether or not they participate in this study. For those that can't quit, the best thing that they can do is reduce the amount that they smoke. The participants will have the opportunity to benefit by making behavioral changes in their smoking, by stopping smoking, or by reducing their exposure to cigarette smoke. Participants who stop smoking will experience invaluable health benefits, and family members of participants who stop smoking would be expected to benefit from reduced/non exposure to second-hand smoke. Participants will be compensated for their time. We will reimburse participants with $50 ClinCards issued by the Research Institute for each assessment for a maximum total of $200 per participant. Participants will be informed that disbursement of the incentives is not contingent on their smoking status.

Importance of the Knowledge to be Gained

Despite over two decades of intensive tobacco control efforts, one in five Americans continue to smoke. Thus, to address the tobacco use epidemic and its consequent health impact, there is an urgent need to test novel interventions for tobacco dependence. The knowledge to be gained from this research could potentially change

the treatment approach offered to smokers with COPD. The risks involved in gaining this knowledge are reasonable given the potential impact of the knowledge to be gained on smoking cessation treatment.

Data Safety and Monitoring Plan

The data safety and monitoring plan for this study follows the guidelines established by the University of Kansas Medical Center Human Research Protection Program. The purpose of this data and safety monitoring plan is to ensure the safety of study participants and the validity of data in compliance with federal requirements for Data and Safety Monitoring for Clinical Trials. Dr. Ellerbeck will report on data safety and monitoring activities as part of an annual progress report to the Human Subjects Committee of the University of Kansas Medical Center as well to PCORI. This section outlines essential elements of the Data Safety and Monitoring Plan for this clinical trial.

In order to assure the accuracy and the security of data, we will adhere to the following data management activities related to data entry, data cleaning, identifying and tagging any crossovers, conversion into proper format for data analysis and recoding. We will use REDCap, a secure, web-based application for building and managing online surveys and databases. A computer based tracking system will be developed to follow each patient and to prompt the staff for the upcoming data collection point. Data collection points for each subject will be calculated from his or her initial date of contact. Data entry will be performed on site under direction of Drs. Ellerbeck and Phadnis. Keypunching routines will adhere to the codebook specifications written by Drs. Ellerbeck and Phadnis. Codebooks will include variable formats (numeric/alpha), min/max ranges and any skip patterns. Both the databases and the tracking system will be password protected for security and maintenance of confidentiality. At the end of each data entry period, data will be backed up onto a secure storage unit. Checks built into the database will ensure that individuals not meeting eligibility criteria are flagged and excluded from data analysis. Standard cleaning and management routines (e.g. conversion of birth dates to ages, logical checks for continuous variables, compliance with skip patterns, missing data codes, etc.) will be conducted using SAS v 9.1.

Our Data Safety and Monitoring Plan will be overseen by a protocol-specific Data Safety and Monitoring Board whose activities, in turn, will be overseen by the KUMC Data Safety and Monitoring Executive Committee. The protocol-specific Data Safety and Monitoring Board will consist of James Vacek, MD, MS-CR, (DSMB chair), a cardiologist with experience in clinical trial oversight, Matthew Sharp, MD, a pulmonologist with experience in management of COPD patients and clinical trials, and John Keighley, PhD, a biostatistician with experience in clinical trial design and safety monitoring..

Dr. Ellerbeck and his research team has met with the DSMB at the outset of the study, prior to subject recruitment and will meet with them annually thereafter. Additional meetings will be held as needed. Quarterly reports including numbers and descriptions of hospitalizations related to heart problems will be reported to the DSMB. The DSMB has/will:

1) Review the study protocols, informed consent and data safety monitoring plan and approve all of these prior to recruitment of subjects.
2) Review the general reporting guidelines based on study procedures approved by the IRB.
3) Approve reporting rules for any known or anticipated adverse outcomes defined broadly for this study protocol.
4) Review plans for data quality and timeliness.
5) Assure adherence to the study protocol, participant recruitment, and consenting procedures.
6) Review and advise the research team on accrual and retention and review adverse events
7) Independently review serious adverse events to determine attribution of the occurrence to the study protocol.
8) Determine when/if an interim analysis is needed and determine if cumulative data indicate the need to change the research design, modify presentation of information to participants, or terminate the project.
9) Review new scientific developments outside of the project that might influence the safety or ethics of the current study.

10) Develop pre-specified stopping rules if significant benefits or risks should develop, if trial management issues prevent successful completion or if compelling ethical issues arise.
11) Evaluate study manuscripts and final reports to assure results are fairly presented and conclusions are appropriate.

Any action taken to suspend or terminate the project by the DSMB will be reported to the KUMC Data Safety and Monitoring Executive Committee and to the PCORI program director.

REFERENCES CITED

1. Centers for Disease C, Prevention. Current cigarette smoking among adults - United States, 2011. MMWR Morbidity and mortality weekly report 2012;61:889-94.
2. Centers for Disease C, Prevention. Smoking-attributable mortality, years of potential life lost, and productivity losses--United States, 2000-2004. MMWR Morbidity and mortality weekly report 2008;57:1226-8.
3. Centers for Disease C, Prevention. Chronic obstructive pulmonary disease among adults--United States, 2011. MMWR Morbidity and mortality weekly report 2012;61:938-43.
4. Rennard S, Decramer M, Calverley PM, et al. Impact of COPD in North America and Europe in 2000: subjects' perspective of Confronting COPD International Survey. The European respiratory journal 2002;20:799-805.
5. Sekine Y, Behnia M, Fujisawa T. Impact of COPD on pulmonary complications and on long-term survival of patients undergoing surgery for NSCLC. Lung cancer 2002;37:95-101.
6. Anthonisen NR, Skeans MA, Wise RA, et al. The effects of a smoking cessation intervention on 14.5-year mortality: a randomized clinical trial. Annals of internal medicine 2005;142:233-9.
7. Makris D, Moschandreas J, Damianaki A, et al. Exacerbations and lung function decline in COPD: new insights in current and ex-smokers. Respiratory medicine 2007;101:1305-12.
8. Scanlon PD, Connett JE, Waller LA, Altose MD, Bailey WC, Buist AS. Smoking cessation and lung function in mild-to-moderate chronic obstructive pulmonary disease. The Lung Health Study. American journal of respiratory and critical care medicine 2000;161:381-90.
9. Jimenez Ruiz CA, Ramos Pinedo A, Cicero Guerrero A, Mayayo Ulibarri M, Cristobal Fernandez M, Lopez Gonzalez G. Characteristics of COPD smokers and effectiveness and safety of smoking cessation medications. Nicotine & tobacco research : official journal of the Society for Research on Nicotine and Tobacco 2012;14:1035-9.
10. Smokers' Own Concern About Smoking Ties Record High. Gallup Poll News Service, 2011. (Accessed July 7, 2013, at http://www.gallup.com/poll/148865/smokers-own-concern-smoking-ties-record-high.aspx.)
11. Shiffman S. Smoking-cessation treatment utilization: The need for a consumer perspective. American journal of preventive medicine 2010;38:S382-4.
12. Hughes JR, Keely J, Naud S. Shape of the relapse curve and long-term abstinence among untreated smokers. Addiction 2004;99:29-38.
13. Fiore M, Jaen C, Baker T, et al. Treating Tobacco Use and Dependence Clinical Practice Guideline: 2008 Update. Washington, DC: U.S. Department of Health and Human Services; 2008.
14. Glasgow RE, Gaglio B, France EK, et al. Do behavioral smoking reduction approaches reach more or different smokers? Two studies; similar answers. Addictive behaviors 2006;31:509-18.
15. Shiffman S, Hughes JR, Ferguson SG, Pillitteri JL, Gitchell JG, Burton SL. Smokers' interest in using nicotine replacement to aid smoking reduction. Nicotine & tobacco research : official journal of the Society for Research on Nicotine and Tobacco 2007;9:1177-82.
16. Stead LF, Lancaster T. Interventions to reduce harm from continued tobacco use. The Cochrane database of systematic reviews 2007:CD005231.
17. Moore D, Aveyard P, Connock M, Wang D, Fry-Smith A, Barton P. Effectiveness and safety of nicotine replacement therapy assisted reduction to stop smoking: systematic review and meta-analysis. Bmj 2009;338:b1024.
18. Shiffman S, Ferguson SG. Nicotine patch therapy prior to quitting smoking: a meta-analysis. Addiction 2008;103:557-63.

19. Wang D, Connock M, Barton P, Fry-Smith A, Aveyard P, Moore D. 'Cut down to quit' with nicotine replacement therapies in smoking cessation: a systematic review of effectiveness and economic analysis. Health technology assessment 2008;12:iii-iv, ix-xi, 1-135.

20. Afonso AM, Ebell MH, Gonzales R, Stein J, Genton B, Senn N. The use of classification and regression trees to predict the likelihood of seasonal influenza. Family practice 2012;29:671-7.

21. Esteban C, Arostegui I, Moraza J, et al. Development of a decision tree to assess the severity and prognosis of stable COPD. The European respiratory journal 2011;38:1294-300.

22. Delaney BC, Fitzmaurice DA, Riaz A, Hobbs FD. Can computerised decision support systems deliver improved quality in primary care?. Interview by Abi Berger. Bmj 1999;319:1281.

23. Sherman CB. Health effects of cigarette smoking. Clinics in chest medicine 1991;12:643-58.

24. Cromwell J, Bartosch WJ, Fiore MC, Hasselblad V, Baker T. Cost-effectiveness of the clinical practice recommendations in the AHCPR guideline for smoking cessation. Agency for Health Care Policy and Research. JAMA : the journal of the American Medical Association 1997;278:1759-66.

25. Shiffman S, Brockwell SE, Pillitteri JL, Gitchell JG. Use of smoking-cessation treatments in the United States. American journal of preventive medicine 2008;34:102-11.

26. Stead LF, Perera R, Bullen C, et al. Nicotine replacement therapy for smoking cessation. The Cochrane database of systematic reviews 2012;11:CD000146.

27. Cahill K, Stevens S, Perera R, Lancaster T. Pharmacological interventions for smoking cessation: an overview and network meta-analysis. The Cochrane database of systematic reviews 2013;5:CD009329.

28. Henningfield JE. Nicotine medications for smoking cessation. The New England journal of medicine 1995;333:1196-203.

29. Sweeney CT, Fant RV, Fagerstrom KO, McGovern JF, Henningfield JE. Combination nicotine replacement therapy for smoking cessation: rationale, efficacy and tolerability. CNS drugs 2001;15:453-67.

30. Burns ME, Bosworth TW, Fiore MC. Insurance coverage of smoking cessation treatment for state employees. American journal of public health 2004;94:1338-40.

31. Levy DE. Employer-sponsored insurance coverage of smoking cessation treatments. The American journal of managed care 2006;12:553-62.

32. Solberg LI, Davidson G, Alesci NL, Boyle RG, Magnan S. Physician smoking-cessation actions: are they dependent on insurance coverage or on patients? American journal of preventive medicine 2002;23:160-5.

33. Hajek P, Stead LF, West R, Jarvis M, Lancaster T. Relapse prevention interventions for smoking cessation. The Cochrane database of systematic reviews 2009:CD003999.

34. Tonstad S, Tonnesen P, Hajek P, et al. Effect of maintenance therapy with varenicline on smoking cessation: a randomized controlled trial. JAMA : the journal of the American Medical Association 2006;296:64-71.

35. How to use Nicoderm CQ. 2013. (Accessed August 2, 2013, at http://www.nicodermcq.com/nicotine-patch/how-to-use.)

36. Hays JT, Leischow SJ, Lawrence D, Lee TC. Adherence to treatment for tobacco dependence: association with smoking abstinence and predictors of adherence. Nicotine & tobacco research : official journal of the Society for Research on Nicotine and Tobacco 2010;12:574-81.

37. Shiffman S, Sweeney CT, Ferguson SG, Sembower MA, Gitchell JG. Relationship between adherence to daily nicotine patch use and treatment efficacy: secondary analysis of a 10-week randomized, double-blind, placebo-controlled clinical trial simulating over-the-counter use in adult smokers. Clinical therapeutics 2008;30:1852-8.

38. Foulds J, Stapleton J, Feyerabend C, Vesey C, Jarvis M, Russell MA. Effect of transdermal nicotine patches on cigarette smoking: a double blind crossover study. Psychopharmacology 1992;106:421-7.
39. Rose JE, Behm FM, Westman EC. Nicotine-mecamylamine treatment for smoking cessation: the role of pre-cessation therapy. Experimental and clinical psychopharmacology 1998;6:331-43.
40. Rose JE, Behm FM, Westman EC, Kukovich P. Precessation treatment with nicotine skin patch facilitates smoking cessation. Nicotine & tobacco research : official journal of the Society for Research on Nicotine and Tobacco 2006;8:89-101.
41. Wennike P, Danielsson T, Landfeldt B, Westin A, Tonnesen P. Smoking reduction promotes smoking cessation: results from a double blind, randomized, placebo-controlled trial of nicotine gum with 2-year follow-up. Addiction 2003;98:1395-402.
42. Nemeth-Coslett R, Henningfield JE. Effects of nicotine chewing gum on cigarette smoking and subjective and physiologic effects. Clinical pharmacology and therapeutics 1986;39:625-30.
43. Fagerstrom KO, Hughes JR. Nicotine concentrations with concurrent use of cigarettes and nicotine replacement: a review. Nicotine & tobacco research : official journal of the Society for Research on Nicotine and Tobacco 2002;4 Suppl 2:S73-9.
44. Nicotine Transdermal Patch. U.S. National Library of Medicine, 2013. (Accessed August 2, 2013, at http://www.nlm.nih.gov/medlineplus/druginfo/meds/a601084.html#precautions.)
45. Benowitz NL. The role of nicotine in smoking-related cardiovascular disease. Preventive medicine 1997;26:412-7.
46. Benowitz NL, Gourlay SG. Cardiovascular toxicity of nicotine: implications for nicotine replacement therapy. Journal of the American College of Cardiology 1997;29:1422-31.
47. Benowitz NL, Porchet H, Sheiner L, Jacob P, 3rd. Nicotine absorption and cardiovascular effects with smokeless tobacco use: comparison with cigarettes and nicotine gum. Clinical pharmacology and therapeutics 1988;44:23-8.
48. Benowitz NL. Clinical pharmacology of nicotine: implications for understanding, preventing, and treating tobacco addiction. Clinical pharmacology and therapeutics 2008;83:531-41.
49. Hwang SH, Pyo T, Oh HB, Park HJ, Lee KJ. Combined application of information theory on laboratory results with classification and regression tree analysis: analysis of unnecessary biopsy for prostate cancer. Clin Chim Acta 2013;415:133-7.
50. Piper ME, Loh WY, Smith SS, Japuntich SJ, Baker TB. Using decision tree analysis to identify risk factors for relapse to smoking. Subst Use Misuse 2011;46:492-510.
51. Loh WY, Piper ME, Schlam TR, et al. Should all smokers use combination smoking cessation pharmacotherapy? Using novel analytic methods to detect differential treatment effects over 8 weeks of pharmacotherapy. Nicotine Tob Res 2012;14:131-41.
52. Hutcheson TD, Greiner KA, Ellerbeck EF, Jeffries SK, Mussulman LM, Casey GN. Understanding smoking cessation in rural communities. The Journal of rural health : official journal of the American Rural Health Association and the National Rural Health Care Association 2008;24:116-24.
53. Ellerbeck EF, Ahluwalia JS, Jolicoeur DG, Gladden J, Mosier MC. Direct observation of smoking cessation activities in primary care practice. The Journal of family practice 2001;50:688-93.
54. Ellerbeck EF, Choi WS, McCarter K, Jolicoeur DG, Greiner A, Ahluwalia JS. Impact of patient characteristics on physician's smoking cessation strategies. Preventive medicine 2003;36:464-70.
55. Cupertino AP, Wick JA, Richter KP, Mussulman L, Nazir N, Ellerbeck EF. The impact of repeated cycles of pharmacotherapy on smoking cessation: a longitudinal cohort study. Archives of internal medicine 2009;169:1928-30.
56. Ellerbeck EF, Mahnken JD, Cupertino AP, et al. Effect of varying levels of disease management on smoking cessation: a randomized trial. Annals of internal medicine 2009;150:437-46.

57. Smoking cessation in rural hospitals. 2013. at http://www.clinicaltrials.gov/ct2/show/NCT01063972?term=Ellerbeck&rank=1.)

58. Faseru B, Turner M, Casey G, et al. Evaluation of a hospital-based tobacco treatment service: outcomes and lessons learned. Journal of hospital medicine : an official publication of the Society of Hospital Medicine 2011;6:211-8.

59. Okuyemi KS, Caldwell AR, Thomas JL, et al. Homelessness and smoking cessation: insights from focus groups. Nicotine & tobacco research : official journal of the Society for Research on Nicotine and Tobacco 2006;8:287-96.

60. Richter KP, Kaur H, Resnicow K, Nazir N, Mosier MC, Ahluwalia JS. Cigarette smoking among marijuana users in the United States. Substance abuse : official publication of the Association for Medical Education and Research in Substance Abuse 2004;25:35-43.

61. Richter KP, McCool RM, Okuyemi KS, Mayo MS, Ahluwalia JS. Patients' views on smoking cessation and tobacco harm reduction during drug treatment. Nicotine & tobacco research : official journal of the Society for Research on Nicotine and Tobacco 2002;4 Suppl 2:S175-82.

62. Ahluwalia JS, Richter K, Mayo MS, et al. African American smokers interested and eligible for a smoking cessation clinical trial: predictors of not returning for randomization. Annals of epidemiology 2002;12:206-12.

63. Jolicoeur DG, Ahluwalia JS, Richter KP, et al. The use of nicotine patches with minimal intervention. Preventive medicine 2000;30:504-12.

64. Jolicoeur DG, Richter KP, Ahluwalia JS, Mosier MC, Resnicow K. Smoking cessation, smoking reduction, and delayed quitting among smokers given nicotine patches and a self-help pamphlet. Substance abuse : official publication of the Association for Medical Education and Research in Substance Abuse 2003;24:101-6.

65. Richter KP, McCool RM, Catley D, Hall M, Ahluwalia JS. Dual pharmacotherapy and motivational interviewing for tobacco dependence among drug treatment patients. Journal of addictive diseases 2005;24:79-90.

66. Catley D, Harris KJ, Goggin K, et al. Motivational Interviewing for encouraging quit attempts among unmotivated smokers: study protocol of a randomized, controlled, efficacy trial. BMC public health 2012;12:456.

67. Cupertino AP, Mahnken JD, Richter K, et al. Long-term engagement in smoking cessation counseling among rural smokers. Journal of health care for the poor and underserved 2007;18:39-51.

68. Cupertino PA, Richter KP, Cox LS, et al. Smoking cessation pharmacotherapy preferences in rural primary care. Nicotine & tobacco research : official journal of the Society for Research on Nicotine and Tobacco 2008;10:301-7.

69. Fawcett SB, Lewis RK, Paine-Andrews A, et al. Evaluating community coalitions for prevention of substance abuse: the case of Project Freedom. Health education & behavior : the official publication of the Society for Public Health Education 1997;24:812-28.

70. Fawcett SB, Paine-Andrews A, Francisco VT, et al. Using empowerment theory in collaborative partnerships for community health and development. American journal of community psychology 1995;23:677-97.

71. Lewis RK, Paine-Andrews A, Fawcett SB, et al. Evaluating the effects of a community coalition's efforts to reduce illegal sales of alcohol and tobacco products to minors. Journal of community health 1996;21:429-36.

72. Paine-Andrews A, Harris KJ, Fawcett SB, et al. Evaluating a statewide partnership for reducing risks for chronic diseases. Journal of community health 1997;22:343-59.

73. Faseru B, Yeh HW, Ellerbeck EE, Befort C, Richter KP. Prevalence and predictors of tobacco treatment in an academic medical center. Joint Commission journal on quality and patient safety / Joint Commission Resources 2009;35:551-7.
74. Ahluwalia JS, Okuyemi K, Nollen N, et al. The effects of nicotine gum and counseling among African American light smokers: a 2 x 2 factorial design. Addiction 2006;101:883-91.
75. Cox LS, Nollen NL, Mayo MS, et al. Bupropion for smoking cessation in African American light smokers: a randomized controlled trial. Journal of the National Cancer Institute 2012;104:290-8.
76. Nollen NL, Mayo MS, Sanderson Cox L, et al. Predictors of quitting among African American light smokers enrolled in a randomized, placebo-controlled trial. Journal of general internal medicine 2006;21:590-5.
77. Buchanan TS, Berg CJ, Cox LS, et al. Adherence to varenicline among African American smokers: an exploratory analysis comparing plasma concentration, pill count, and self-report. Nicotine & tobacco research : official journal of the Society for Research on Nicotine and Tobacco 2012;14:1083-91.
78. Nollen NL, Mayo MS, Ahluwalia JS, et al. Factors Associated with Discontinuation of Bupropion and Counseling Among African American Light Smokers in a Randomized Clinical Trial. Annals of behavioral medicine : a publication of the Society of Behavioral Medicine 2013.
79. National Cancer Institute. Clearing the air. U.S. Department of Health and Human Services 2011.
80. TTS Certification. (Accessed August 3, 2013, at http://www.umassmed.edu/tobacco/training/tts_cert.aspx.)
81. Kornitzer M, Boutsen M, Dramaix M, Thijs J, Gustavsson G. Combined use of nicotine patch and gum in smoking cessation: a placebo-controlled clinical trial. Preventive medicine 1995;24:41-7.
82. Puska PK, H.J.; Vartiainen,E.; Urjanheimo,E.L.; Gustavsson,G.; Westin,A. . Combined use of nicotine patch and gum compared with gum alone in smoking cessation: a clinical trial in North Karelia. Tobacco Control 1995;4:231-325.
83. Glasgow RE, Orleans CT, Wagner EH. Does the chronic care model serve also as a template for improving prevention? The Milbank quarterly 2001;79:579-612, iv-v.
84. Wagner EH, Austin BT, Von Korff M. Organizing care for patients with chronic illness. The Milbank quarterly 1996;74:511-44.
85. Hughes JR, Benowitz N, Hatsukami D, Mermelstein RJ, Shiffman S. Clarification of SRNT workgroup guidelines for measures in clinical trials of smoking cessation therapies. Nicotine & tobacco research : official journal of the Society for Research on Nicotine and Tobacco 2004;6:863-4.
86. Hughes JR, Keely JP, Niaura RS, Ossip-Klein DJ, Richmond RL, Swan GE. Measures of abstinence in clinical trials: issues and recommendations. Nicotine & tobacco research : official journal of the Society for Research on Nicotine and Tobacco 2003;5:13-25.
87. JJones, P. W., et al. (2009). "Development and first validation of the COPD Assessment Test." Eur Respir J **34**(3): 648-654..
88. Hecht SS. Tobacco smoke carcinogens and lung cancer. Journal of the National Cancer Institute 1999;91:1194-210.
89. Hecht SS. Tobacco carcinogens, their biomarkers and tobacco-induced cancer. Nature reviews Cancer 2003;3:733-44.
90. Benowitz NL, Dains KM, Dempsey D, Wilson M, Jacob P. Racial differences in the relationship between number of cigarettes smoked and nicotine and carcinogen exposure. Nicotine & tobacco research : official journal of the Society for Research on Nicotine and Tobacco 2011;13:772-83.
91. Heatherton TF, Kozlowski LT, Frecker RC, Fagerstrom KO. The Fagerstrom Test for Nicotine Dependence: a revision of the Fagerstrom Tolerance Questionnaire. Br J Addict 1991;86:1119-27.

92. Littel RCM, G.A.; Stroup,W.W.; Wolfinger,R.D.; Schabenberger,O. SAS for mixed models. Cary, NC: SAS Institute Inc.; 2007.

93. Hedeker D, Mermelstein RJ, Demirtas H. Analysis of binary outcomes with missing data: missing = smoking, last observation carried forward, and a little multiple imputation. Addiction 2007;102:1564-73.

94. Breiman LF, J.; Stone,C.J.; Olshen,R.A. Classification and Regression Trees. Boca Raton, FL: CRC Press; 1984.

95. Therneau TMA, E.J. An Introduction to Recursive Partitioning Using RPART Routines. Rochester, MN: Mayo Foundation; 1997.

96. Classification and Regression Trees, Data Mining Lecture Notes. 2009. (Accessed August 7, 2013, at www.stat.cmu.edu/~cshalizi/350/lectures/22/lecture-22.pdf.)

97. National Priorities for Research and Research Agenda. PCORI, 2012. (Accessed August 4, 2013, at http://www.pcori.org/assets/PCORI-National-Priorities-and-Research-Agenda-2012-05-21-FINAL1.pdf.)

98. Hammond D, Reid JL, Driezen P, et al. Smokers' use of nicotine replacement therapy for reasons other than stopping smoking: findings from the ITC Four Country Survey. Addiction 2008;103:1696-703.

99. Backinger CL, Thornton-Bullock A, Miner C, et al. Building consumer demand for tobacco-cessation products and services: The national tobacco cessation collaborative's consumer demand roundtable. American journal of preventive medicine 2010;38:S307-11.

100. Shiffman S, Cone EJ, Buchhalter AR, et al. Rapid absorption of nicotine from new nicotine gum formulations. Pharmacology, biochemistry, and behavior 2009;91:380-4.

101. Richter KP, Arnsten JH. A rationale and model for addressing tobacco dependence in substance abuse treatment. Substance abuse treatment, prevention, and policy 2006;1:23.

102. Richter KP, Hamilton AK, Hall S, Catley D, Cox LS, Grobe J. Patterns of smoking and methadone dose in drug treatment patients. Experimental and clinical psychopharmacology 2007;15:144-53.

103. Richter KP, Hunt JJ, Cupertino AP, Garrett S, Friedmann PD. Understanding the drug treatment community's ambivalence towards tobacco use and treatment. The International journal on drug policy 2012;23:220-8.

104. Chen Y, Rennie DC, Dosman JA. The reliability of cigarette consumption reports by spousal proxies. A J Public Health. 1995; 85(12):1711-12.

105. Smith SS, Piper ME, Bolt DM, Fiore MC, Wetter DW, Cinciripini PM, Baker TB. Development of the brief Wisconsin inventory of smoking dependence motives. Nicotine Tob Res. 2010 May;12(5):489-99.
