# Supplementary material for: Effect of Long-term Nicotine Replacement Therapy vs Standard Smoking Cessation for Smokers With Chronic Lung Disease: A Randomized Clinical Trial
Source: JAMA Netw Open. 2018 Sep 7;1(5):e181843. doi: 10.1001/jamanetworkopen.2018.1843 (PMC6324503; doi:10.1001/jamanetworkopen.2018.1843)
Supplement: Supplement 2. — eTable 1. Bivariate and Multivariate Associations Between Participant Characteristics and CO-Verified 7-Day Point Prevalence of Abstinence at Month 12 eTable 2. Bivariate and Multivariate Associations Between Participant Characteristics and NNAL Reduction From Baseline to Month 12 eTable 3. Bivariate and Multivariate Associations Between Participant Characteristics and 50% Reduction in CO From Baseline to Month 12 [file jamanetwopen-1-e181843-s002.pdf]

Supplementary Online Content

Ellerbeck EF, Nollen N, Hutcheson TD, et al. Effect of long-term nicotine replacement therapy vs standard smoking cessation for smokers with chronic lung disease: a randomized clinical trial. *JAMA Netw Open.* 2018;1(5):e181843. doi:10.1001/jamanetworkopen.2018.1843

eTable 1. Bivariate and Multivariate Associations Between Participant Characteristics and CO-Verified 7-Day Point Prevalence of Abstinence at Month 12
eTable 2. Bivariate and Multivariate Associations Between Participant Characteristics and NNAL Reduction From Baseline to Month 12
eTable 3. Bivariate and Multivariate Associations Between Participant Characteristics and 50% Reduction in CO From Baseline to Month 12

This supplementary material has been provided by the authors to give readers additional information about their work.

© 2018 Ellerbeck EF et al. *JAMA Network Open*.

eTable 1. Bivariate and Multivariate Associations Between Participant Characteristics and CO-Verified 7-Day Point Prevalence of Abstinence at Month 12

	Bivariate Analysis		Multivariate Analysis	
**Variable**	**OR (95% CI)**	**p-value**	**OR (95% CI)**	***P* Value**
Study arm (LT-NRT=1, SSC=0)	1.05 (0.57-1.93)	.88	--	--
Age, years (≥60 =1, <60=0)	4.55 (2.40-8.62)	< .001	4.30 (2.25-8.19)	<.001
Gender (Male=1, Female=0)	1.81 (0.98-3.33)	.06	--	--
Race (African American=1, Other=0)[a]	0.84 (0.41-1.74)	.64	--	--
Employed (Yes=1, No=0)	1.65 (0.83-3.31)	.16	--	--
Medicaid (Yes=1, No=0)	0.67 (0.35-1.26)	.21	--	--
Other smoker in home (Yes=1, No=0)	0.70 (0.37-1.34)	.28	--	--
Smoking allowed in the home (Yes=1, No=0)	0.78 (0.37-1.67)	.54		
Nicotine dependence (Heavy=1, Light/Moderate=0)[b]	0.66 (0.36-1.23)	.19	--	--
Cotinine[c]	0.99 (0.87-1.15)	.95	--	--
Nicotine Metabolite Ratio (Fast/Normal Metabolizer=1, Slow=0)[d]	0.94 (0.51-1.74)	.85	--	--
COPD symptoms (<20=1, ≥20=0)[e]	1.88 (1.02-3.46)	.04	1.57 (0.83-2.96)	.17
COPD Exacerbation in past year (Yes=1, No=0)	0.82 (0.43-1.58)	.56	--	--
Respiratory Function[f]	0.97 (0.84-1.14)	.75	--	--
Anxiety (≥3=1, <3=0)[g]	1.80 (0.93-3.47)	.08	--	--
Depressive symptoms (≥3=1, <3=0)[h]	1.54 (0.77-3.08)	.22	--	--
Planning to quit in next 30 days (Yes=1, No=0)	0.94 (0.43-2.04)	.87	--	--
Confidence to quit[i]	1.10 (0.99-1.25)	.09	--	--
Daily NRT use at Month 12[j] (Yes=1, No=0)	1.53 (0.83-2.83)	.18	--	--

bbreviations: CO, carbon monoxide; OR, odds ratio; CI, confidence interval; LT-NRT, long-term nicotine replacement therapy; SSC, standard smoking cessation; COPD, chronic obstructive pulmonary disease; NRT, nicotine replacement therapy

For all participants (n=394), participant characteristic variables were added to a bivariate logistic regression model to examine their association with CO-verified 7-day point prevalence abstinence at Month 12 (n=47 participants were abstinent at Month 12). Any factors with p<.05 were added to a multivariate regression model.

[a]Race: Other includes American Indian or Alaska Native, Asian, Native Hawaiian or Other Pacific Islander, Caucasian.
[b]Heaviness of Smoking Index scores range from 0 to 6. Scores of 4 or greater indicate high nicotine dependence.
[c]OR reflects 1000 unit increase in cotinine.
[d]Nicotine Metabolite Ratio=(3-hydroxycotinine/cotinine), log-transformed. Mixed model-based cut-point of bimodal distribution (1.45) was used to categorize slow metabolizers vs. normal/fast metabolizers.
[e]COPD Assessment Test scores range from 0 to 40. Score of 20 or more indicates high symptoms.
[f]OR reflects 10 unit increase in FEV1.
[g]General Anxiety Disorder-2 scores range from 0 to 6. Scores of 3 or greater indicate presence of general anxiety disorder. [h]Patient Health Questionnaire-2 scores range from 0 to 6. Scores of 3 or greater indicate potential presence of depression.
[i]OR reflects 1 unit increase in confidence. Confidence scores range from 1 to 10, with 10 indicating higher levels of confidence.
[j]Daily NRT use at M12 was defined as using one or more types of NRT each day for the past 3 days.

© 2018 Ellerbeck EF et al. *JAMA Network Open*.

eTable 2. Bivariate and Multivariate Associations Between Participant Characteristics and NNAL Reduction From Baseline to Month 12

**Variable**	**Bivariate Analysis**			**Multivariate Analysis**	
	**β (95% CI)**	**Exp(β)[a] (95% CI)**	**p-value**	**Exp(β)[a] (95% CI)**	**p-value**
Study arm (LT-NRT=1, SSC=0)	0.05 (-0.13 to 0.23)	1.05 (0.88 to 1.26)	.56	--	--
Age, years (≥60=1, <60=0)	-0.03 (-0.23 to 0.17)	0.97 (0.80 to 1.18)	.76	--	--
Gender (Male=1, Female=0)	0.09 (-0.09 to 0.28)	1.10 (0.91 to 1.32)	.33	--	--
Race (African American=1, Other=0)[b]	0.03 (-0.19 to 0.26)	1.03 (0.82 to 1.29)	.78	--	--
Employed (Yes=1, No=0)	0.02 (-0.22 to 0.27)	1.02 (0.80 to 1.31)	.85	--	--
Medicaid (Yes=1, No=0)	-0.08 (-0.27 to 0.10)	0.92 (0.77 to 1.10)	.36	--	--
Other smoker in home (Yes=1, No=0)	0.15 (-0.03 to 0.33)	1.16 (0.97 to 1.40)	.11	--	--
Smoking allowed in the home (Yes=1, No=0)	-0.12 (-0.36 to 0.12)	0.89 (0.70 to 1.12)	.31		
Nicotine dependence (Heavy=1, Light/Moderate=0)[c]	0.00 (-0.19 to 0.18)	1.00 (0.83 to 1.20)	.98	--	--
Cotinine[d]	-0.04 (-0.09 to -0.001)	0.96 (0.92 to 0.999)	.04	0.80 (0.67 to 0.97)	.04
NMR (Fast/Normal Metabolizer=1, Slow=0)[e]	-0.08 (-0.26 to 0.10)	0.92 (0.77 to 1.10)	.37	--	--
COPD symptoms (<20=1, ≥20=0)[f]	-0.01 (-0.20 to 0.18)	0.99 (0.82 to 1.19)	.90	--	--
COPD Exacerbation in past year (Yes=1, No=0)	-0.03 (-0.22 to 0.16)	0.97 (0.81 to 1.17)	.76	--	--
Respiratory Function[g]	0.00 (-0.05 to 0.04)	1.00 (0.96 to 1.04)	.96	--	--
Anxiety (≥3=1, <3-0)[h]	-0.11 (-0.29 to 0.08)	0.90 (0.75 to 1.08)	.25	--	--
Depressive symptoms (≥3=1, <3-0)[i]	-0.22 (-0.40 to -0.03)	0.80 (0.67 to 0.97)	.02	0.82 (0.68 to 0.98)	.02
Planning to quit in next 30 days (Yes=1, No=0)	0.01 (-0.23 to 0.24)	1.01 (0.79 to 1.28)	.96	--	--
Confidence to quit[j]	-0.01 (-0.05 to 0.02)	0.99 (0.96 to 1.02)	.44	--	--
Daily NRT use at Month 12[k] (Yes=1, No=0)	-0.19 (-0.38 to -0.01)	0.82 (0.68 to 0.99)	.04	0.96 (0.92 to 1.00)	.03

Abbreviations: NNAL, 4-(methylnitrosamino)-1-(3-pyridyl)-1-butanol; CI, confidence interval; LT-NRT, long-term nicotine replacement therapy; SSC, standard smoking cessation; NMR, Nicotine Metabolic Ratio; COPD, chronic obstructive pulmonary disease; NRT, nicotine replacement therapy; FEV1, forced expiratory volume in 1 second

For all continuing smokers at Month 12 with NNAL data at Baseline and Month 12 (n=310), participant characteristic variables were added to a bivariate linear regression model to examine their association with NNAL reduction from Baseline to Month 12. Any factors with p<.10 were added to a multivariate regression model.

[a]Change in NNAL was calculated on the log-scale, so the Beta coefficient and 95% CI were exponentiated. The Exp(β) expresses the multiplicative effect on the NNAL value per unit change in the covariate.

[b]Race: Other includes American Indian or Alaska Native, Asian, Native Hawaiian or Other Pacific Islander, Caucasian.

[c]Heavy Smoking Index scores range from 0 to 6. Scores of 4 or greater indicate high nicotine dependence.

[d]Exp(β) reflects 1000 unit increase in cotinine.

[e]Nicotine Metabolite Ratio=(3-hydroxycotinine/cotinine), log-transformed. Mixed model-based cut-point of bimodal distribution (1.45) was used to categorize slow metabolizers vs normal/fast metabolizers.

[f]COPD Assessment Test scores range from 0 to 40. Score of 10 or more indicates high symptoms (GOLD 2017).

[g]Exp(β) reflects 10 unit increase in FEV1.

[h]General Anxiety Disorder-2 scores range from 0 to 6. Scores of 3 or greater indicate presence of general anxiety disorder.

[i]Patient Health Questionnaire-2 scores range from 0 to 6. Scores of 3 or greater indicate potential presence of depression.

[j]Exp(β)reflects 1 unit increase in confidence. Confidence scores range from 1 to 10, with 10 indicating higher levels of confidence.

[k]Daily NRT use at Month 12 was defined as using one or more types of NRT each day for the past 3 days.

eTable 3. *Bivariate and multivariate relationships between participant characteristics and 50% reduction in CO from Baseline to Month 12*.

© 2018 Ellerbeck EF et al. *JAMA Network Open*.

eTable 3. Bivariate and Multivariate Associations Between Participant Characteristics and 50% Reduction in CO From Baseline to Month 12

eTable 3. *Bivariate and multivariate relationships between participant characteristics and 50% reduction in CO from Baseline to Month 12*.

	Bivariate Analysis		Multivariate Analysis	
**Variable**	**OR (95% CI)**	**p-value**	**OR (95% CI)**	**p-value**
Study arm (LT-NRT=1, SSC=0)	1.42 (0.85-2.37)	.18	--	--
Age, years (≥60=1, <60=0)	1.11 (0.64-1.95)	.69	--	--
Gender (Male=1, Female=0)	1.25 (0.74-2.10)	.40	--	--
Race (African American=1, Other=0)[a]	0.63 (0.34-1.16)	.14	--	--
Employed (Yes=1, No=0)	1.90 (0.85-4.26)	.12	--	--
Medicaid (Yes=1, No=0)	1.34 (0.80-2.23)	.26	--	--
Other smoker in home (Yes=1, No=0)	1.76 (1.05-2.95)	.03	1.38 (0.88-2.17)	.16
Smoking allowed in the home (Yes=1, No=0)	1.08 (0.54-2.15)	.82	--	--
Nicotine dependence (Heavy=1, Light/Moderate=0)[b]	0.65 (0.39-1.10)	.11	--	--
Cotinine[c]	0.88 (0.76-1.01)	.07	1.00 (1.00-1.00)	.76
Nicotine Metabolite Ratio (Fast/Normal Metabolizer=1, Slow=0)[d]	1.72 (1.03-2.88)	.04	1.31 (0.77-2.26)	.31
COPD symptoms (<20=1, ≥20=0)[e]	0.65 (0.38-1.14)	.13	--	--
COPD Exacerbation in past year (Yes=1, No=0)	1.57 (0.94-2.64)	.09	1.32 (0.83-2.08)	.24
Respiratory Function[f]	0.88 (0.77-1.001)	.05	0.99 (0.98-1.004)	.22
Anxiety (≥3=1, <3-0)[g]	0.71 (0.43-1.19)	.20	--	--
Depressive symptoms (≥3=1, <3-0)[h]	0.83 (0.49-1.42)	.50	--	--
Planning to quit in next 30 days (Yes=1, No=0)	1.70 (0.81-3.57)	.16	--	--
Confidence to quit[i]	0.96 (0.88-1.06)	.41	--	--
Daily NRT use at Month 12[j] (Yes=1, No=0)	1.06 (0.63-1.80)	.82	--	--

Abbreviations: CO, carbon monoxide; OR, odds ratio; CI, confidence interval; LT-NRT, long-term nicotine replacement therapy; SSC, standard smoking cessation; COPD, chronic obstructive pulmonary disease; NRT, nicotine replacement therapy; FEV1, forced expiratory volume in 1 second

For all continuing smokers at Month 12 with CO data at Baseline and Month 12 (n=305), participant characteristic variables were added to a bivariate logistic regression model to examine their association with a 50% reduction in CO from Baseline to Month 12 (n=80 participants had a 50% reduction in CO). Any factors with p<.10 were added to a multivariate regression model.

[a]Race: Other includes American Indian or Alaska Native, Asian, Native Hawaiian or Other Pacific Islander, Caucasian.

[b]Heaviness of Smoking Index scores range from 0 to 6. Scores of 4 or greater indicate high nicotine dependence.

[c]OR reflects 1000 unit increase in cotinine.

[d]Nicotine Metabolite Ratio=(3-hydroxycotinine/cotinine), log-transformed. Mixed model-based cut-point of bimodal distribution (1.45) was used to categorize slow metabolizers vs normal/fast metabolizers.

[e]COPD Assessment Test scores range from 0 to 40. Score of 20 or more indicates high symptoms.

[f]OR reflects 10 unit increase in FEV1.

[g]General Anxiety Disorder-2 scores range from 0 to 6. Scores of 3 or greater indicate presence of general anxiety disorder.

[h]Patient Health Questinnaire-2 scores range from 0 to 6. Scores of 3 or greater indicate potential presence of depression.

[i]OR reflects 1 unit increase in confidence. Confidence scores range from 1 to 10, with 10 indicating higher levels of confidence.

© 2018 Ellerbeck EF et al. *JAMA Network Open*.

[j]Daily NRT use at Month 12 was defined as using one or more types of NRT each day for the past 3 days.

© 2018 Ellerbeck EF et al. *JAMA Network Open*.
